# Reliability and acceptance of dreaMS, a software application for people with multiple sclerosis: a feasibility study

**DOI:** 10.1007/s00415-022-11306-5

**Published:** 2022-08-30

**Authors:** Tim Woelfle, Silvan Pless, Oscar Reyes, Andrea Wiencierz, Anthony Feinstein, Pasquale Calabrese, Konstantin Gugleta, Ludwig Kappos, Johannes Lorscheider, Yvonne Naegelin

**Affiliations:** 1 Department of Neurology and MS Center, University Hospital and University of Basel, Basel, Switzerland; 2 Research Center for Clinical Neuroimmunology and Neuroscience Basel, Basel, Switzerland; 3 Healios AG, Basel, Switzerland; 4 Clinical Trial Unit, Department of Clinical Research, University Hospital, University of Basel, Basel, Switzerland; 5 Department of Psychiatry, Sunnybrook Health Sciences Centre, Toronto, Canada; 6 Neuropsychology and Behavioral Neurology Unit, Division of Molecular and Cognitive Neuroscience, University of Basel, Basel, Switzerland; 7 Ophthalmology, University Hospital Basel and University of Basel, Basel, Switzerland; 8 Department of Neurology, University Hospital Basel, Petersgraben 4, 4031 Basel, Switzerland

**Keywords:** Multiple sclerosis, Digital biomarkers, Smartphone, Smartwatch, Mobile health

## Abstract

**Background:**

There is an unmet need for reliable and sensitive measures for better monitoring people with multiple sclerosis (PwMS) to detect disease progression early and adapt therapeutic measures accordingly.

**Objective:**

To assess reliability of extracted features and meaningfulness of 11 tests applied through a smartphone application (“dreaMS”).

**Methods:**

PwMS (age 18–70 and EDSS ≤ 6.5) and matched healthy volunteers (HV) were asked to perform tests installed on their smartphone once or twice weekly for 5 weeks. Primary outcomes were test–retest reliability of test features (target: intraclass correlation [ICC] ≥ 0.6 or median coefficient of variation [mCV] < 0.2) and reported meaningfulness of the tests by PwMS. Meaningfulness was self-assessed for each test on a 5-point Likert scale (target: mean score of > 3) and by a structured interview. ClinicalTrials.gov Identifier: NCT04413032.

**Results:**

We included 31 PwMS (21 [68%] female, mean age 43.4 ± 12.0 years, median EDSS 3.0 [range 1.0–6.0]) and 31 age- and sex-matched healthy volunteers. Out of 133 features extracted from 11 tests, 89 met the preset reliability criteria. All 11 tests were perceived as highly meaningful to PwMS.

**Conclusion:**

The dreaMS app reliably assessed features reflecting key functional domains meaningful to PwMS. More studies with longer follow-up are needed to prove validity of these measures as digital biomarkers in PwMS.

**Supplementary Information:**

The online version contains supplementary material available at 10.1007/s00415-022-11306-5.

## Introduction

Multiple sclerosis (MS) is a chronic inflammatory demyelinating disease of the central nervous system leading to a broad variety of neurological symptoms and increasing disability over time [1, 2]. Movement, balance, dexterity, cognition, vision, fatigue, sensory, and autonomic functions are major disease domains typically impaired by MS and traditionally quantified by overall assessment tools such as the Expanded Disability Status Scale (EDSS) and its functional systems [3, 4], as well as tests capturing specific domains, such as the Timed 25-Foot Walk (T25FW, short distance ambulation speed) [5]; Nine Hole Peg Test (9-HPT, dexterity of upper extremities) [6] and Symbol Digit Modalities Test (SDMT, cognitive processing speed) [7]. Tests like these are useful in clinical practice, but objective assessment of additional and more detailed features of disease-related functional impairment, such as balance or step regularity, could add valuable information. More importantly, more frequent or even continuous non-intrusive assessment in the patients’ respective natural environment would complement the relatively infrequent assessments in clinical practice. In the era of the COVID-19 pandemic, remote assessment in the natural environment of people with MS (PwMS) became even more important.

App-based tests can use smartphone sensors to capture objective, quantifiable measures of physiology and/or behavior. Algorithms transform them into features, also called digital measures, which in turn might serve as digital biomarkers once validated for complementary clinical value to traditional measures [8]. Recent reviews highlight the potential benefits of digital assessments [9, 10] and digital therapeutics [11], and several industry-led initiatives to develop digital biomarkers for PwMS are underway (e.g., Konectom and Floodlight [12, 13]).

The smartphone app “dreaMS” is developed in the framework of an academically led project to create and validate generally accepted digital outcomes. The objective of this feasibility study was to determine test–retest reliability of features captured through 11 active tests included in dreaMS and to assess their acceptance and perceived meaningfulness to PwMS.

## Methods

The study protocol was approved by the local ethics committee (Ethikkommission Nordwest- und Zentralschweiz (EKNZ), Basel, Switzerland, on July 17th 2020/project-ID 2020–01,515). All participants gave their written informed consent. This study conforms with World Medical Association Declaration of Helsinki and was registered at ClinicalTrials.gov: NCT04413032.

### Participants

PwMS and age- and sex-matched healthy volunteers (HV) were included into this feasibility study, performed at the MS Clinic, University Hospital Basel from October 5th, 2020, to February 28th, 2021. Inclusion criteria for both PwMS and HV were: age 18–70, possession of a dreaMS compatible smartphone (iOS ≥ 11.0/Android ≥ 5.0), hand motor skills sufficient for using a smartphone, corrected binocular near visual acuity of ≥ 0.5, being able to walk without aid, ability and willingness to follow the study procedures. PwMS additional inclusion criteria were: diagnosis of MS according to the revised McDonald criteria 2017 [14] irrespective of disease course, EDSS ≤ 6.5, being in a stable phase of their disease at the time of inclusion and throughout the 6 weeks of study duration confirmed by two Neurostatus-EDSS examinations. As this study focused on the reliability of the measures, PwMS who experienced a relapse or clinical progression during the study (defined as any EDSS worsening at the end-of-study visit compared to baseline) were excluded. Exclusion criteria for both PwMS and HV were: other clinically significant concomitant diseases, known or suspected non-compliance, drug or alcohol abuse, and pregnancy or breast feeding.

No formal sample size calculation was performed for this first feasibility study due to the two-step project setup with a larger validation study currently underway (NCT05009160). The pre-planned sample size (30 PwMS and 30 HV) was estimated based on published results of similar research in the field [15, 16].

### Study procedures

Study duration was 6 weeks per participant, and 3 study visits were performed: screening, baseline, and end-of-study. At the screening visit, written informed consent was obtained from all participants. At the baseline visit, demographic data were collected. PwMS underwent a standardized Neurostatus-EDSS examination [3], and all participants performed T25FW, 9-HPT, and SDMT. Best corrected near visual acuity was assessed using a validated 40 cm chart [17] and low contrast vision with a Pelli-Robson Trans-Illuminated Contrast Sensitivity Chart for Low Vision and Peak CS with Landolt C optotypes for an ETDRS Illuminator Cabinet (Model 2425E) [18] at three meters distance. Additionally, a consumer wearable device (smartwatch Fitbit Versa 2™) was handed out to all participants at baseline and was connected to their smartphone. Data collected by this device were accessed securely through the Fitbit web application programming interface. The study participants were asked to wear the smartwatch continuously during the study. At the end-of-study visit, Neurostatus-EDSS was repeated to confirm clinical stability and user feedback was collected by questionnaires and a structured interview.

Participants were asked to repeatedly perform 11 tests over the first 5 weeks of the study at home using the dreaMS app that was installed on their smartphones (iOS or Android) at baseline. They were carefully instructed by a study nurse to ensure that they performed all tests correctly. Data collected by smartphone sensors (accelerometer, gyroscope, and magnetometer at 50 Hz as well as touchscreen usage) during the performance of these tests were transferred to a secure cloud data base and processed through algorithms which transform the continuous sensor data into features. An overview of the 11 tests and examples of extracted features are shown in Table 1. A full list of all extracted features is provided in Table S1. Example screenshots of the app can be found in Supplementary Figure S3. In the 6th week of the study, patient reported outcome measures (PROMs) were obtained within the dreaMS app: Fatigue Severity Scale [19, 20], MSIS-29 (The Multiple Sclerosis Impact Scale) [21], and MSWS-12 (Twelve Item MS Walking Scale) [22].

**Table 1:** Overview of domains, tests and examples of extracted features

Domain	Test	Short description of test	Examples of extracted features
Movement	U-Turn	Repeatedly make a U-turn after 5 steps taken for 30 s	Number of U-turns, mean turn time, mean angular velocity
	Two-minute walk	Walk briskly for 2 min, without break	Number of steps, mean step time, mean step regularity
	Climbing stairs	Walk up and down a set of stairs, indicate number of steps	Number of steps, mean step time, mean step regularity
	Musical chairs	Repeatedly stand-up and sit-down on a chair for 30 s	Number of repetitions, mean time to sit down, mean complete move time
Balance	Arm balance	Keep arms up for 10 s each, first with eyes open and then again with eyes closed	Normalized path length, total power accelerometer, mean velocity
	Standing balance	Stand with arms along the body for 10 s, first with eyes open, then again with eyes closed	Normalized path length, jerk sway
Dexterity	Screen-to-nose	Hold phone in hand and bend arm such that the tip of the nose touches the bulls eye on the screen, with both arms, eyes open and closed (15 s each)	Number of green touches, mean distance, mean bend time
	Catch-a-cloud	Touch the moving cloud with index finger as often as possible in 30 s	Number of successful touches, number of total touches, mean distance
Cognition	m-SDMT	Call out the correct number orally for the presented symbols using a key in 8 consecutive trials (adaptation of Feinstein’s auto-SDMT)	Mean response time, number of successful responses in 1st-8th trial/all trials
Vision	Acuity	Near visual acuity, for both eyes (swipe in direction of tumbling E)	Acuity fraction
	Low contrast vision	Low contrast acuity, for both eyes (swipe in direction of Landolt C)	Pelli-Robson log contrast sensitivity

The dreaMS app and its in-app tests were designed together with Healios AG, Switzerland, our technical development partner and manufacturer. For cognition, an adaptation of the auto-SDMT [23] was implemented into dreaMS (without demographic information and integrated vision tests). As the fully automated speech recognition did not work sufficiently, mp3-audio files were analyzed manually.

DreaMS also provided a deep link to selected adaptive cognitive games of a commercial app covering main domains of cognition expected to be compromised in PwMS [24]; results to be reported separately.

### Objectives, outcomes & statistical analysis

The *primary objective*s were to identify features that are both measurable and technically reliable, and to select tests that are user-friendly and meaningful for PwMS. Hence, *the primary study outcomes* were (a) reliability of features across all scheduled test repetitions as defined by an intraclass correlation (ICC) ≥ 0.6 [25] or by a median coefficient of variation (mCV) ˂ 0.2 [26]; and (b) user acceptance and meaningfulness of tests as reported on a questionnaire (˃ 3 on a 5-point Likert scale). Ten repetitions were scheduled for movement, balance, and dexterity tests (twice weekly), and 5 for cognition and vision tests (once weekly). Reliability was calculated from all available test repetitions. Some tests consisted of several subtests (e.g., left hand/right hand, eyes open/eyes closed), in which case the ICC and mCV were calculated separately for each of those subtests. Every test fulfilling the above-mentioned criteria for at least one feature was selected. For the primary analysis, outliers were identified stratified by user, feature, and subtest (if applicable) and were excluded based on Tukey’s fences [27], with the rationale that they most likely reflect errors in test performance (e.g., a dropped smartphone or distraction of the user). A sensitivity analysis including outliers was also performed. 95% confidence intervals (CI) provided for ICC were derived from bootstrapping with 1000 repetitions. Additionally, we assessed differences between platforms (iOS/Android) with Wilcoxon rank-sum tests as “platform separability”. Undesirable systematic platform differences were assumed if absolute (unsigned) rank-biserial correlation (|*r*|) between iOS and Android was ≥ 0.4.

We assessed the following *exploratory outcomes*: a) Correlations of subject-wise median test features with established functional reference tests for their respective domains (e.g., “Catch-a-cloud” and 9-HPT), the EDSS, and PROMs were calculated using Spearman correlation. 95% CIs were calculated using the Bonett & Wright correction [28]. (b) Separability of subject-wise median test features between PwMS and HV on a group level was tested with Wilcoxon rank-sum tests and reported as rank-biserial correlation r. For each test, we selected a best-performing feature based on reliability and exploratory analyses as outlined above.

Smartwatch usability was assessed as a post hoc* additional exploratory analysis*. Mean proportion of time per day during which heart-rate data from the device was available per user (mean-daily-wear-fraction) was assessed as a marker of adherence. Group differences for standard smartwatch features were assessed with Wilcoxon rank-sum tests. Activity lasting at least 10 min above 3 metabolic equivalents (METs) was defined as “fairly active” and above 6 METs as “very active” [29].

### Data sharing

The data that support the findings of this study are available from the corresponding author upon reasonable request.

### Funding

This work was supported by the Swiss Innovation Agency (Innosuisse, project-ID 33535.1 IP-ICT).

## Results

31 PwMS (21 [68%] female, mean age 43.4 ± 12.0 years, median EDSS 3.0 [range 1.0–6.0]) and 31 age- and sex-matched HVs (21 [68%] female, mean age 42.8 ± 11.9 years) were included in this study (see Table 2 for a detailed description of the baseline characteristics). In total, 31 different smartphone models were used (14 iOS and 17 Android). All participants completed the study according to the protocol, and all PwMS remained stable without relapses or signs of progression (as confirmed by EDSS at baseline and end-of-study). No serious adverse events occurred. One person participated at baseline visit despite a recent relapse, who was considered a screening failure and excluded from all analyses.

**Table 2:** Baseline characteristics of participants

	PwMS (*n* = 31)	HV (*n* = 31)
Female, *n* (%)	21 (68%)	21 (68%)
Mean age ± std	43.4 ± 12.0	42.8 ± 11.9
Disease course, *n* (%)	CIS: 2 (6%) RRMS: 23 (74%) SPMS: 2 (6%) PPMS: 4 (13%)	N/A
Test completion, median (range)	100% (50.8–100%)	98.9% (69.5–100%)
Smartphone platform, *n* (%)	iOS: 21 (68%) Android: 10 (32%)	iOS: 21 (68%) Android: 10 (32%)
9-hole-peg-test time dominant hand, seconds, median (range)	19 (14–31)	17 (14–28)
9-hole-peg-test time non-dominant hand, seconds, median (range)	22 (15–63)	19 (15–28)
25-foot walk test, seconds, median (range)	4 (3–17)	4 (2–6)
Mean SDMT score ± std	57.9 ± 14.5	61.1 ± 11.1
Median EDSS (range)	3.0 (1.0–6.0)	N/A
Disease-modifying treatment, *n*, (%)	Untreated: 6 (19%) Interferon beta-1a: 1 (3%) Glatiramer acetate 1 (3%) Teriflunomide: 3 (10%) Dimethyl fumarate: 1 (3%) Fingolimod: 7 (22%) Natalizumab: 2 (6%) Rituximab: 1 (3%) Ocrelizumab: 9 (29%)	N/A

### Primary outcomes

#### Reliability

In the main analysis, 89 of 133 features (66.9%) extracted from the 11 tests met the target criteria of ICC ≥ 0.6 or mCV < 0.2, see Fig. S1 and Table S2. In the sensitivity analysis, which included outliers, 72 out of 133 features (54.1%) were selected, see Table S3. ICC, mCV, and platform separability are presented in Table 3 for the best-performing features, the complete list for all features is shown in Table S4. Platform separability |r| among the best-performing features was ranging from 0.00 to 0.59 (lower numbers being better) and was below our cutoff of 0.4 for at least one subtest for all tests except Catch-a-cloud.

**Table 3:**
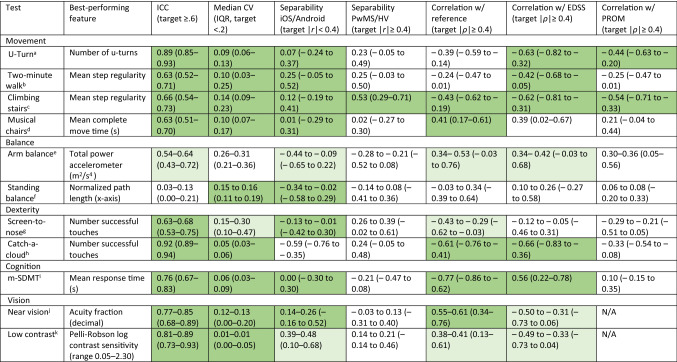
Best-performing feature per test

Features are unitless if not otherwise stated. Brackets contain 95% CIs if not otherwise stated. Ranges show minimum and maximum of all respective subtests

*ICC* intraclass correlation coefficient, *CV* coefficient of variation

^a^U-Turn: Reference: T25FW (minimum), PROM: MSWS

^b^Two-minute walk: Reference: T25FW (minimum), PROM: MSWS

^c^Climbing stairs: Reference: T25FW (minimum), PROM: MSIS-29 Physical Subscore

^d^Musical chairs: Reference: T25FW (minimum), PROM: MSIS-29 Physical Subscore

^e^Arm balance: Reference: Romberg subscore, PROM: MSIS-29 Physical Subscore, 4 subtests: right hand eyes open/closed; left hand eyes open/closed

^f^Standing balance: Reference: Romberg subscore, PROM: MSIS-29 Physical Subscore, 2 subtests: eyes open/closed

^g^Screen-to-nose: Reference: 9-HPT (minimum), PROM: MSIS-29 Physical Subscore, 4 subtests: right hand eyes open/closed; left hand eyes open/closed

^h^Catch-a-cloud: Reference: 9-HPT (minimum), PROM: MSIS-29 Psychological Subscore

^i^m-SDMT: Reference: SDMT, PROM: MSIS-29 Psychological Subscore

^j^Near vision: Reference: Tumbling E Near Chart (40 cm), 2 subtests: right/left eye

^k^Low contrast: Reference: Pelli-Robson Contrast Sensitivity Chart, 2 subtests: right/left eye

Similarity iOS/Android: 1—effect size of Mann–Whitney *U* test of iOS vs. Android (as absolute rank-biserial correlation); Correlation: absolute Spearman correlation coefficient of median top feature per test (after outlier removal) with clinical reference test (see subtext)/EDSS/PROM; green: feature meets target; light green: feature meets target in at least one subtest

#### Meaningfulness

All 11 tests were perceived as meaningful by PwMS and met the predefined target (≥ 3) with mean Likert scale scores between 4.27 (*Standing balance*) and 4.67 (*Arm balance*). Overall impression and willingness to perform the tests in the future was good (mean score 4.09 ± 0.23 and 3.82 ± 0.21, respectively). For detailed meaningfulness and acceptance scores, see Table 4; for exact wording of the questions, see Table S5.

**Table 4:**
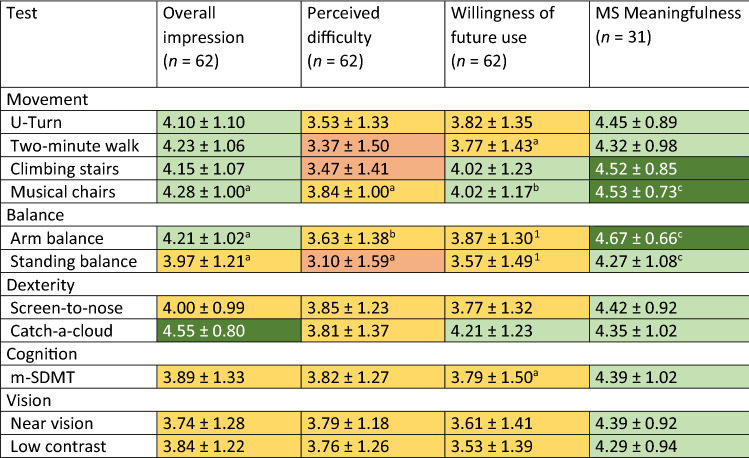
Meaningfulness and user acceptance by mean ± SD Likert scale response (1–5)

Red 3.00–3.50, Yellow 3.50–4.00, Light green 4.00–4.50, dark green 4.50–5.00

^a^*n* = 61

^b^*n* = 60

^c^*n* = 30

All 11 tests met our adherence target of ≥ 80% with a mean percentage of completed tests of 96% (range 91%-99%).

### Exploratory outcom*es*

#### Correlations of Test features with established assessments

**Fig. 1:**
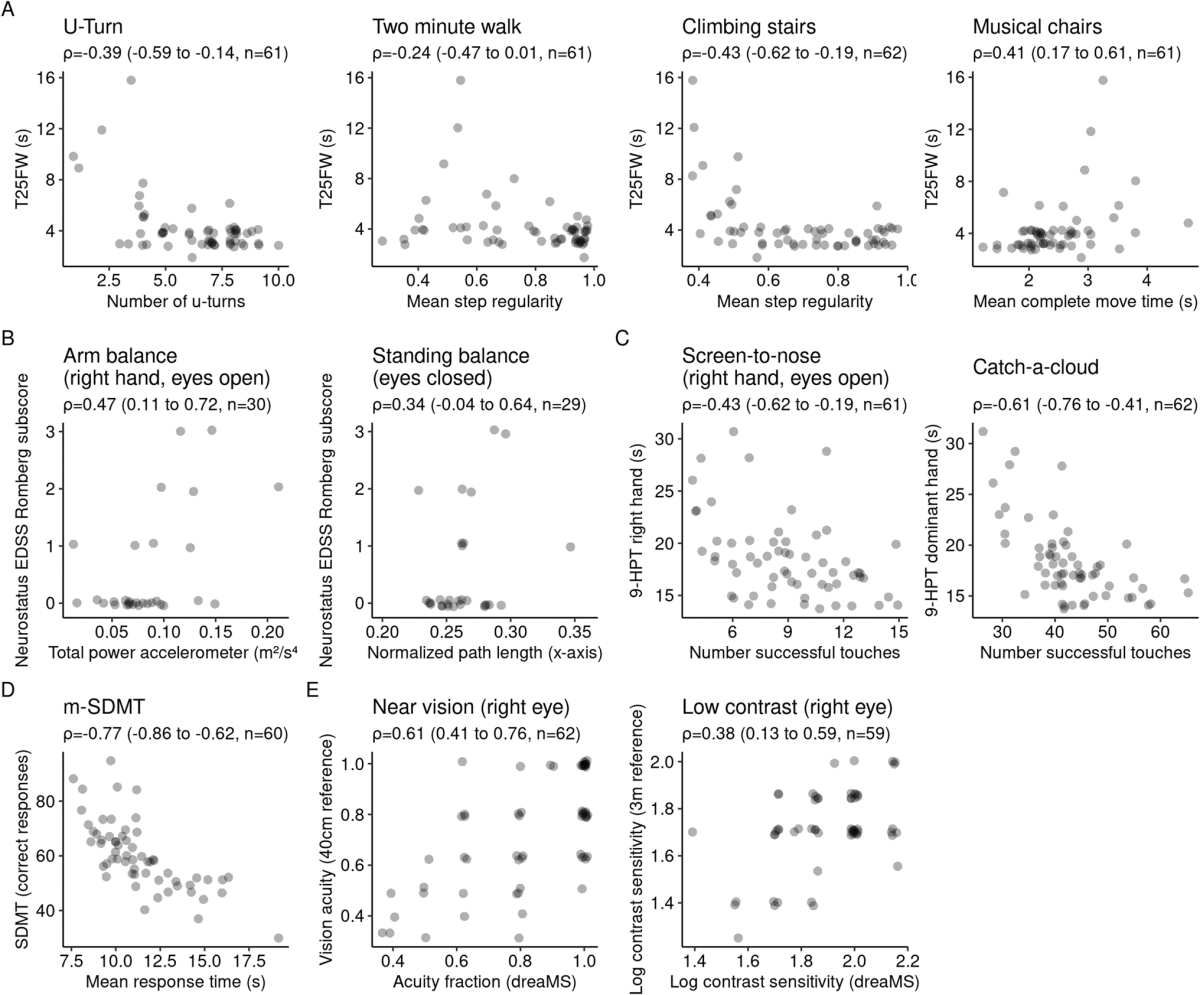
Correlation of top features with clinical reference tests: Spearman ρ (with 95% CI) for top features of **(A)** movement tests, **(B)** balance tests (only among PwMS), **(C)** dexterity tests, **(D)** m-SDMT, and **(E)** vision tests

Correlations of the best-performing features with the respective preselected reference tests ranged from 0.03 to 0.77 (see Fig. 1 and Table 3). Most 95% CIs did not include 0, except for the correlation of *Two-minute walk* with T25FW (*ρ* =  −0.24, 95% CI − 0.47 to 0.01), and *Standing balance* with Neurostatus-EDSS Romberg subscore (*ρ* = 0.34, 95% CI − 0.04 to 0.64). The strongest correlation between test feature and clinical reference test was observed between *Mean response time* in the *m-SDMT* and the number of correct responses within 90 s of the classical SDMT (Spearman *ρ* = −0.77). An overall similar picture was seen when analyzing correlations of the best-performing features with the EDSS (absolute range 0.01–0.66); 95% CI not including the indifference value 0 except for *Arm balance*, *Standing balance*, *Screen-to-nose*, *Near vision*, and *Low contrast*. The strongest correlation with the EDSS was observed for the dexterity assessment *Catch-a-cloud* (*Number of successful touches*, *ρ* = –0.66)*,* followed by *U-Turn* (*Number of U-turns*, *ρ* =  −0.63), *Climbing stairs* (*Mean step regularity*, *ρ* = −0.62), and *Two-minute walk* (*Mean step regularity*, *ρ* = −0.42). The best-performing features from *Climbing stairs* and *U-Turn* also showed the closest correlations with the respective PROM, the MSWS-12 total score (*ρ* = −0.54 and −0.44 respectively). Overall absolute correlations of reliable features with PROMs were ranging from 0.06 to 0.54. The 95% CIs were not including 0 for *U-Turn*, *Climbing stairs*, *Arm balance*, and *Catch-a-cloud* (see Table 3).

#### Comparison of test features between PwMS and HV on a group level

The rank-biserial correlation between PwMS and healthy volunteers of the best-performing features ranged between |*r*|= 0.03 and 0.57 (Table 3) with most CIs including 0. The best group separability was achieved by the *Mean step regularity* feature from the Climbing stairs test (*r* = 0.53; 95% CI 0.29–0.71).

### Additional exploratory analysis of smartwatch usability

For 7 out of 62 users (11%), no smartwatch data (Fitbit Versa 2) were available at all because of problems with device synchronization. Mean-daily-wear-fraction of the remaining 28 PwMS was 94.1% (median; IQR 86.4–95.9%, range 57.2–97.7%) and of the remaining 27 HV 93.3% (median; IQR 87.8%-96.1%, range 21.5%-97.8%). PwMS had shorter median proportions during which they were “fairly active” or “very active” during the day compared to HV (proportion fairly active: 0.3% (IQR 0.0–1.0%) vs 0.8% (IQR 0.4–1.5%), respectively, rank-biserial *r* = 0.37, 95% CI 0.08–0.60; proportion very active: 0.4% (IQR 0.0–0.8%) vs 1.1% (IQR 0.5–1.7%), respectively, *r* = 0.50, 95% CI 0.23–0.69). Similarly, the estimated median daily energy consumption as measured in METs (metabolic equivalents) was lower for PwMS vs HV (21,632 METs 23,128 METs, r = 0.31, 95% CI 0.02–0.56), as well as the median number of steps (7188 steps for PwMS vs 10,338 steps for HV, *r* = 0.51, 95% CI 0.24–0.70) and estimated median walking distance per day (5.3 km for PwMS vs 7.3 km for HV, *r* = 0.48, 95% CI 0.21–0.68; Table S6 and Supplementary Fig. S2).

## Discussion

With the availability of more and increasingly efficient treatments with different mechanisms of action and differing risk–benefit ratios, the need for sensitive and reliable measures of disease activity and progression—a prerequisite of individualized therapeutic decisions—becomes more urgent. Neurological examination and established standardized assessment scales like the EDSS or MSFC capture selected aspects of function but neglect many relevant features of disease-related limitations. Smartphones and smartwatches provide options for comprehensive, frequent to quasi-continuous, and objective measurement of key neurological functions affected by MS in the patient’s natural environment.

In this feasibility study, we focused on the assessment of test–retest reliability of active tests covering 5 functional domains typically affected by MS (movement, balance, dexterity, cognition, and vision) applied through dreaMS, a smartphone application specifically designed for PwMS. We showed that they allow for reliable, unsupervised, remote measurement of neurologic functions in the “natural” patient environment. The features extracted from these tests met the predefined reliability criteria. Importantly, PwMS rated all tests highly for user friendliness and meaningfulness.

Out of the 133 features extracted from all 11 tests, 89 (after exclusion of outliers; 72 when outliers were included) proved to be reliable and all of the 11 tests produced at least one reliable feature. The criterion chosen to define feature reliability was a combination of ICC and CV in order to overcome their respective shortcomings: ICC is known to be low in homogeneous groups, even when test–retest reliability is high, as it is based on the proportion of the between-group variance over total variance. On the other side, ICC tends to be high in heterogeneous groups even when the heterogeneity stems from systematic bias, such as platform separability. CV is more robust in this respect but does not incorporate between-group variance at all. Reflecting the different characteristics of these measures, slightly more features met the mCV target (*n* = 75) than the ICC target (*n* = 63).

Reported data on reliability of other smartphone applications with similar scope are scarce or not directly comparable. For Konectom, only data from one repetition 30 days apart were reported, both done under medical supervision [12]. For Floodlight, the aggregated reliability of daily tests over multiple two-week windows was reported [13]. This approach reduces within-group variance and thus may inflate the assessment of reliability. In addition, we must take into account that all other studies presented up to now used one selected “standard” device that was provided to study participants (e.g., Samsung S7 in [13]). With the aim of reflecting the real world situation as closely as possible, participants in our study used their own devices (31 different models).

Keeping these differences in mind, most ICCs in our study were comparable to those reported for similar tests in Floodlight [13]: 0.76 for dreaMS *m-SDMT* vs 0.55-0.85 for Floodlight *e-SDMT*, 0.92 for dreaMS *Catch-a-cloud* vs 0.71-0.81 for Floodlight *Pinching test*, 0.89 for dreaMS *U-Turn* vs 0.45-0.83 for Floodlight *U-Turn*, and 0.63 for dreaMS *Two-minute walk* vs 0.78-0.85 for Floodlight *Two-minute walk*.

All 11 tests were perceived as meaningful among PwMS. Lower popularity was seen for *m-SDMT* and for the two vision tests, most probably attributable to issues with user experience that in the meantime have been resolved.

The high adherence (96%; range 91–99%) should be interpreted with caution as the study lasted only 6 weeks per participant and longer studies have shown severe drops in adherence [30]. In addition to automated reminders of the test schedule within the dreaMS app, the study nurse called the participants if tests were not performed as scheduled.

This study was not designed for analyzing correlations with functional reference tests, the EDSS, or PROMs, but the exploratory analyses showed promising correlations for all tests except *Standing balance*. The study was also not designed and powered to detect group differences between PwMS and HV. In the exploratory analyses, *Climbing stairs* was the only test whose features differentiated significantly between PwMS and HV on a group level, which might be explained by the overall low disease severity of patients (median EDSS 3.0). *Climbing stairs* thus seems to be among the most sensitive tests to detect subtle gait pathology.

The additional exploratory analysis of the data obtained through passive monitoring with the commercially available smartwatch Fitbit Versa 2™ showed very high adherence levels with a median subject-wise mean-daily-wear-fraction of 93.7%. The standard smartwatch features step count, walking distance, and daily energy consumption separated PwMS and HVs on the group level, supporting the high potential of passive monitoring. Moreover, passive monitoring best reflects spontaneous, everyday function and thus may ensure complementary ecological validity in addition to monitoring through active tests like those used in dreaMS.

In a recent study, motor features detected through passive monitoring with an accelerometer were better correlated with PROMs than with the EDSS [16], while our motor features correlated better with the EDSS than with PROMs. This apparent discrepancy may result from the fact that the active tests included in our app focus at specified deficits in neurological function, which are also the focus of neurological measures like the EDSS or T25FW. Passive monitoring as in the study by Abbadessa et al. reflects the respective everyday activity level, which is dependent on several other factors besides specific neurological deficits. Similarly, PROMs are also dependent on other factors like mood or environment.

Limitations of our study include the low number of participants not reflecting the full range of impairment, especially regarding higher disability. Given the voluntary nature of study participation, we cannot exclude a selection bias that may have favored the high meaningfulness reported by the participants. Additionally, the relatively short study duration did not allow the assessment of long-term adherence and long-term practice effects, which have been described for mobile tests of cognition and dexterity [31]. Finally, the algorithms used for feature extraction and for detection of outliers unrelated to the disease need further improvement.

Overall, the observed reliability and high grade of perceived meaningfulness of the tests included in dreaMS encourage further development of this software application as an important future monitoring tool for PwMS. As with other proposed applications for monitoring MS, longer-term studies, for example in well-documented large cohorts of PwMS, must follow to support sensitivity to change and concurrent as well as content validity as digital biomarkers [32]. A first validation study is currently recruiting (NCT05009160).

## Supplementary Information

Below is the link to the electronic supplementary material.

Supplementary file1 (DOCX 1053 KB)
