# Supplementary material for: Reliability and acceptance of dreaMS, a software application for people with multiple sclerosis: a feasibility study
Source: J Neurol. 2022 Aug 30;270(1):262–71. doi: 10.1007/s00415-022-11306-5 (PMC9427170; doi:10.1007/s00415-022-11306-5)

Reliability and Acceptance of dreaMS, a Software Application for People with Multiple Sclerosis – a Feasibility Study

Tim Woelfle, Silvan Pless, Oscar Reyes, Andrea Wiencierz, Anthony Feinstein, Pasquale Calabrese, Konstantin Gugleta, Ludwig Kappos, Johannes Lorscheider^*✉^, Yvonne Naegelin^*^

* equally contributing last authors

^✉^ corresponding author: [johannes.lorscheider@usb.ch](mailto:johannes.lorscheider@usb.ch)

Supplementary Information

[Table S1: Description of all 133 features 1](#_Toc108714622)

[Table S2: Reliability summary results – main analysis, outliers excluded 9](#_Toc108714623)

[Table S3: Reliability summary results – sensitivity analysis, outliers included 9](#_Toc108714624)

[Table S4: Reliability results by extracted feature 10](#_Toc108714625)

[Table S5: Acceptance questionnaire 18](#_Toc108714626)

[Table S6: Smartwatch median daily features in HV and PwMS 19](#_Toc108714627)

[Figure S1: Reliability overview: ICC vs mCV for all features and subtests 20](#_Toc108714628)

[Figure S2: Smartwatch median daily features in n=27 HV and n=28 PwMS 21](#_Toc108714629)

[Figure S3: Example screenshots from the dreaMS app version 1 22](#_Toc108714630)

## Table S1: Description of all 133 features

| **Test** | **Feature (digital measure)** | **Description** | **Unit** |
| --- | --- | --- | --- |
| U-Turn | num_uturns | The number of u-turns performed. | unitless |
|  | mean_uturn_time | Average time to perform a u-turn. | seconds |
|  | std_uturn_time | Standard deviation of the time to perform a uturn. | seconds |
|  | mean_angular_velocity | Average angular velocity to perform a u-turn. | grad/s |
|  | std_angular_velocity | Standard deviation of the velocity to perform a u-turn. | grad/s |
| Two minute walk | num_steps | Number of steps estimated from signal. | unitless |
|  | mean_step_time | Average time of one step. | seconds |
|  | cadence | Gait cadence; the number of steps per time unit. | steps/sec |
|  | step_time_variability | Step time variability that represents the standard deviation of the step times. | seconds |
|  | step_time_coef_variation | Coefficient of variation of time steps. | unitless (percentage) |
|  | mean_step_reg | Average step regularity. An unbiased autocorrelation procedure was used to measure the correlation of the acceleration signal for each step (first dominant period). Step regularity is defined as the correlation between the original acceleration signals of each pair of steps. | unitless (correlation) |
|  | mean_even_step_reg | Similar to the feature mean_step_reg, but only considering even steps. | unitless (correlation) |
|  | mean_odd_step_reg | Similar to the feature mean_step_reg, but only considering odd steps. | unitless (correlation) |
|  | mean_step_dtw | Average similarity, or regularity, between pair of steps calculated by using Dynamic Time Warping (DTW) measure. DTW is a measure used for determining an optimal alignment between two time series/signals, and between its advantages are the ability to compare sequences which differ in length and its generality. | unitless (score) |
|  | mean_even_step_dtw | Similar to mean_step_dtw, but only considering the even steps. | unitless (score) |
|  | mean_odd_step_dtw | Similar to mean_step_dtw, but only considering the odd steps. | unitless (score) |
|  | mean_step_jerksw | Average JerkSw feature calculated on the segments of the signal representing each step. Jerk swayness indicates the change in the movement smoothness, and it is calculated via integration of the sum of second derivates of changes in acceleration in medio lateral and antero posterior axes. | m^2/s^5 |
|  | mean_even_step_jerksw | Average JerkSw feature calculated on the segments of the signal representing each even step. | m^2/s^5 |
|  | mean_odd_step_jerksw | Average JerkSw feature calculated on the segments of the signal representing each odd step. | m^2/s^5 |
|  | num_strides | The number of strides performed (left + right step or vice-versa). | unitless |
|  | mean_stride_time | Average time stride. | seconds |
|  | stride_time_variability | Stride time variability; standard deviation of the required time for one stride. | seconds |
|  | stride_time_coef_variation | The same logic like step time variability (coefficient of variation), but calculated over stride times. | unitless (percentage) |
|  | mean_stride_reg | Similar to step regularity, stride regularity was defined as the correlation between the original acceleration signals of each pair of strides. | unitless (correlation) |
|  | mean_stride_dtw | Average DTW similarity score between strides. | unitless (score) |
|  | mean_stride_jerksw | Average JerkSw feature calculated on the segments of the signal representing each stride. | m^2/s^5 |
|  | gait_sym | Gait symmetry; it represents the percent difference between the regularity of steps and the regularity of strides. | unitless (percentage) |
|  | jerksw_complete_signal | JerkSw feature calculated on the whole signal. | m^2/s^5 |
|  | num_steps_pedometer | Number of steps recorded by pedometer. | unitless |
|  | apple_total_pedometer_list | Distance estimated by pedometer. | meters |
|  | apple_mean_estimated_step_length | Average step length estimated from apple_total_estimated_dist and num_steps. | meters |
|  | apple_mean_estimated_stride_length | Average length of strides estimated from apple_total_estimated_dist and num_steps. | meters |
|  | apple_total_estimated_dist | Distance estimated from num_steps, num_steps_pedometer, and apple_total_pedometer_list. | meters |
| Climbing stairs | num_stairs | Number of stairs climbed. This value is extracted from the survey conducted at the end of the exercise. | unitless |
|  | num_steps | Number of steps estimated from the accelerometer signal. | unitless |
|  | mean_step_time | Average time for one step (i.e., right and left combined). | seconds |
|  | cadence | Gait cadence; the number of steps per time unit (steps/seconds). | steps/sec |
|  | step_time_variability | Step time variability that represents the standard deviation of the step times. | seconds |
|  | step_time_coef_variation | Coefficient of variation of time steps. | unitless (percentage) |
|  | mean_step_reg | Average step regularity. An unbiased autocorrelation procedure was used to measure the correlation of the acceleration signal for each step (first dominant period). Step regularity is defined as the correlation between the original acceleration signals of each pair of steps. | unitless (correlation) |
|  | mean_even_step_reg | Similar to the feature mean_step_reg, but only considering even steps. | unitless (correlation) |
|  | mean_odd_step_reg | Similar to the feature mean_step_reg, but only considering odd steps. | unitless (correlation) |
|  | mean_step_dtw | Average similarity, or regularity, between pair of steps calculated by using Dynamic Time Warping (DTW) measure. DTW is a measure used for determining an optimal alignment between two time series/signals, and between its advantages are the ability to compare sequences which differ in length and its generality. | unitless (score) |
|  | mean_even_step_dtw | Similar to mean_step_dtw, but only considering the even steps. | unitless (score) |
|  | mean_odd_step_dtw | Similar to mean_step_dtw, but only considering the odd steps. | unitless (score) |
|  | mean_step_jerksw | Average JerkSw feature calculated on the segments of the signal representing each step. Jerk swayness indicates the change in the movement smoothness, and it is calculated via integration of the sum of second derivates of changes in acceleration in medio lateral and antero posterior axes. | m^2/s^5 |
|  | mean_even_step_jerksw | Average JerkSw feature calculated on the segments of the signal representing each even step. | m^2/s^5 |
|  | mean_odd_step_jerksw | Average JerkSw feature calculated on the segments of the signal representing each odd step. | m^2/s^5 |
|  | num_strides | Number of strides performed (left + right step or vice-versa). | unitless |
|  | mean_stride_time | Average time of each set of subsequent left and right steps together. It is approximately equal to the double of mean step time. | seconds |
|  | stride_time_variability | Stride time variability; standard deviation of the required time for one stride. | seconds |
|  | stride_time_coef_variation | The same logic like step time variability (coefficient of variation), but calculated over stride times. | unitless (percentage) |
|  | mean_stride_reg | Similar to step regularity, stride regularity was defined as the correlation between the original acceleration signals of each pair of strides. | unitless (correlation) |
|  | mean_stride_dtw | Average DTW similarity score between strides. | unitless (score) |
|  | mean_stride_jerksw | Average JerkSw feature calculated on the segments of the signal representing each stride. | m^2/s^5 |
|  | gait_sym | Gait symmetry; it represents the percent difference between the regularity of steps and the regularity of strides. | unitless (percentage) |
|  | jerksw_complete_signal | JerkSw feature calculated on the whole signal. | m^2/s^5 |
| Musical chairs | num_sitting | Number of squads. | unitless |
|  | mean_sitting_time | Average time to sit. | seconds |
|  | std_sitting_time | Average standard deviation of times to sit. | seconds |
|  | mean_sitting_velocity | Average velocity to sit. | m/s |
|  | std_sitting_velocity | Standard deviation of velocity to sit. | m/s |
|  | mean_sitting_jerksw | Average jerk swayness to sit. Jerk swayness indicates the change in the movement smoothness, and it is calculated via integration of the sum of second derivates of changes in acceleration in medio lateral and antero posterior axes. | m^2/s^5 |
|  | std_sitting_jerksw | Standard deviation of JerkSw feature to sit. | m^2/s^5 |
|  | mean_standing_time | Average time to stand up. | seconds |
|  | std_standing_time | Standard deviation of times to stand up. | seconds |
|  | mean_standing_velocity | Average velocity to stand up. | m/s |
|  | std_standing_velocity | Standard deviation of velocity to stand up. | m/s |
|  | mean_standing_jerksw | Average JerkSw to stand up. | m^2/s^5 |
|  | std_standing_jerksw | Standard deviation of JerkSw feature to stand up. | m^2/s^5 |
|  | mean_complete_move_time | Average time to complete a movement (sit + stand up). | seconds |
|  | std_complete_move_time | Standard deviation of time to complete a movement. | seconds |
| Arm balance | npl_ml | Normalized path length in medio-lateral direction. The normalized path length (NPL) represents the normalised sum of displacement of the hand holding the phone in the medio-lateral axis. It summarises how much the user have moved the hand in this direction. | unitless |
|  | npl_v | Normalized path length in vertical (V) direction. Similar to npl_v but in V direction. | unitless |
|  | npl_ap | Normalized path length in anterior posterior (AP) direction. Similar to npl_ml but in AP direction. | unitless |
|  | mean_velocity_ml | Mean velocity of the phone in medio lateral axis. | m/s |
|  | mean_velocity_v | Mean velocity of the phone in vertical axis. | m/s |
|  | mean_velocity_ap | Mean velocity of the phone in antero posterior axis. | m/s |
|  | rms_tremor_gyr | Root mean square (RMS) of the gyroscope data in the axis of greater amplitude. It represents the RMS tremor in gyroscope signal. | rad/s |
|  | total_power_gyr | Represents the average level of tremor, calculated by using the gyroscope, that the user expressed during the challenge. | rad^2/s^2 |
|  | peak_power_gyr | Represents the maximum level of tremor, calculated by using the gyroscope, that the user expressed during the challenge. | rad^2/s^2 |
|  | rms_tremor_acc | Root mean square (RMS) of the accelerometer data in the axis of greater amplitude. It represents the RMS kinetic tremor in accelerometer signal. | m/s^2 |
|  | total_power_acc | Represents the average level of tremor, calculated by using the accelerometer, that the user expressed during the challenge. | m^2/s^4 |
|  | peak_power_acc | Represents the maximum level of tremor, calculated by using the accelerometer, that the user expressed during the challenge. | m^2/s^4 |
| Standing balance | npl_ml | Normalized path length in medio-lateral direction. The normalized path length (NPL) indicates a typical deviation around a center of mass and it is used as indicator for quantifying postural stability of patients with cerebellar disorders. It represents the normalized sum of absolute differences of positions in the medio lateral axis. | unitless |
|  | npl_ap | Normalized path length in antero-posterior direction. Similar to npl_ml but in the medio lateral axis. | unitless |
|  | jerksw | Jerk swayness indicates the change in the movement smoothness, and it has been used as sway scores to measure balance problems or postural stability. JerkSw is calculated via integration of the sum of second derivates of changes in acceleration in medio lateral and antero posterior axes. | m^2/s^5 |
| Screen-to-nose | num_green_touches | Number of touches on the screen within the center of the bullseye. | unitless |
|  | num_yellow_touches | Number of touches on the screen within the bounds of the bullseye. | unitless |
|  | num_total_touches | Total number of touches on the screen (successful + failed touches). | unitless |
|  | mean_mm_distance | Average distance (in millimetres) between the touches on the screen and the center of the bullseye. | mm |
|  | std_mm_distance | Standard deviation of the distance (in millimetres) between the touches on the screen and the center of the bullseye. | mm |
|  | num_bends | Times that the user touch the screen of the smartphone with his nose. | unitless |
|  | mean_bend_time | Average time to bend the arm. | seconds |
|  | std_bend_time | Standard deviation time to bend the arm. | seconds |
|  | mean_stretch_time | Average time to stretch the arm. | seconds |
|  | std_stretch_time | Standard deviation time to stretch the arm. | seconds |
|  | mean_bend_jerksw | Average value of Jerk swayness while bending the arm. Jerk swayness indicates the change in the movement smoothness, and it is calculated via integration of the sum of second derivates of changes in acceleration in medio lateral and antero posterior axes. | m^2/s^5 |
|  | std_bend_jerksw | Standard deviation value of Jerk swayness while bending the arm. | m^2/s^5 |
|  | mean_stretch_jerksw | Average value of Jerk swayness while stretching the arm. | m^2/s^5 |
|  | std_stretch_jerksw | Standard deviation value of Jerk swayness while stretching the arm. | m^2/s^5 |
|  | mean_bend_rms_tremor_acc | Average value of root mean square of the accelerometer data while bending the arm. It represents the RMS kinetic tremor in accelerometer signal. | m/s^2 |
|  | std_bend_rms_tremor_acc | Standard deviaton value of root mean square of the accelerometer data while bending the arm. It represents the RMS kinetic tremor in accelerometer signal. | m/s^2 |
|  | mean_stretch_rms_tremor_acc | Average value of root mean square of the accelerometer data while stretching the arm. | m/s^2 |
|  | std_stretch_rms_tremor_acc | Standard deviation value of root mean square of the accelerometer data while stretching the arm. | m/s^2 |
|  | mean_bend_rms_tremor_gyr | Average value of root mean square of the gyroscope data while bending the arm. It represents the RMS kinetic tremor in gyroscope signal. | rad/s |
|  | std_bend_rms_tremor_gyr | Standard deviation value of root mean square of the gyroscope data while bending the arm. It represents the RMS kinetic tremor in gyroscope signal. | rad/s |
|  | mean_stretch_rms_tremor_gyr | Average value of root mean square of the gyroscope data while stretching the arm. | rad/s |
|  | std_stretch_rms_tremor_gyr | Standard deviation value of root mean square of the gyroscope data while stretching the arm. | rad/s |
|  | mean_bend_velocity | Average value of velocity while bending the arm. | m/s |
|  | std_bend_velocity | Standard deviation of the velocity while bending the arm. | m/s |
|  | mean_stretch_velocity | Average value of velocity while stretching the arm. | m/s |
|  | std_stretch_velocity | Standard deviation of velocity while stretching the arm. | m/s |
|  | mean_full_movement_time | Average time to complete a full movement (bend + stretch the arm). | seconds |
|  | std_full_movement_time | Standard deviation time to complete a full movement (bend + stretch the arm). | seconds |
| Catch-a-cloud | num_successful_touches | Number of touches representing when the user touched the cloud successfully. | unitless |
|  | num_total_touches | Number of total touches (successful + failed touches). | unitless |
|  | mean_mm_distance | Average of the distance (in millimetres) between the user's touches and the cloud's position. | mm |
|  | std_mm_distance | Standard deviation of the distance between the user's touches and the cloud's position. | mm |
| m-SDMT | correct_responses_trial_1 | Number of successful responses by the user in the first trial. | unitless |
|  | correct_responses_trial_2 | Number of successful responses by the user in the second trial. | unitless |
|  | correct_responses_trial_3 | Number of successful responses by the user in the third trial. | unitless |
|  | correct_responses_trial_4 | Number of successful responses by the user in the fourth trial. | unitless |
|  | correct_responses_trial_5 | Number of successful responses by the user in the fifth trial. | unitless |
|  | correct_responses_trial_6 | Number of successful responses by the user in the sixth trial. | unitless |
|  | correct_responses_trial_7 | Number of successful responses by the user in the seventh trial. | unitless |
|  | correct_responses_trial_8 | Number of successful responses by the user in the eighth trial. | unitless |
|  | total_correct_responses | Total number of successful responses in all trials. | unitless |
|  | mean_time_response | Average time between user's responses. | seconds |
| Near vision | final_score_left_eye | Final score achieved by the user with the left eye. | unitless |
|  | final_score_right_eye | Final score achieved by the user with the right eye. | unitless |
| Low contrast | final_score_left_eye | Final score achieved by the user with the left eye. | unitless |
|  | final_score_right_eye | Final score achieved by the user with the right eye. | unitless |

## Table S2: Reliability summary results – main analysis, outliers excluded

| **Test** | **Total number of features** | **Features with ICC ≥ 60%** | **Features with mCV < 20%** | **Number of features fulfilling the criteria for reliability** |
| --- | --- | --- | --- | --- |
| U-Turn | 5 | 1 | 3 | 3 |
| Two minute walk | 28 | 7 | 19 | 20 |
| Climbing stairs | 24 | 18 | 15 | 19 |
| Musical chairs | 15 | 2 | 4 | 5 |
| Arm balance | 12 | 3 | 4 | 6 |
| Standing balance | 3 | 1 | 2 | 2 |
| Screen-to-nose | 28 | 16 | 10 | 16 |
| Catch-a-cloud | 4 | 2 | 4 | 4 |
| m-SDMT | 10 | 9 | 10 | 10 |
| Near vision | 2 | 2 | 2 | 2 |
| Low contrast | 2 | 2 | 2 | 2 |
| **Sum** | **133** | **63** | **75** | **89** |

## Table S3: Reliability summary results – sensitivity analysis, outliers included

| **Test** | **Total number of features** | **Feature ICC ≥ 60%** | **Feature mCV < 20%** | **Number of features selected** |
| --- | --- | --- | --- | --- |
| U-Turn | 5 | 1 | 3 | 3 |
| Two minute walk | 28 | 1 | 14 | 14 |
| Climbing stairs | 24 | 8 | 15 | 17 |
| Musical chairs | 15 | 0 | 3 | 3 |
| Arm balance | 12 | 1 | 3 | 4 |
| Standing balance | 3 | 0 | 2 | 2 |
| Screen-to-nose | 28 | 10 | 7 | 12 |
| Catch-a-cloud | 4 | 2 | 3 | 3 |
| m-SDMT | 10 | 1 | 10 | 10 |
| Near vision | 2 | 2 | 2 | 2 |
| Low contrast | 2 | 2 | 2 | 2 |
| **Sum** | **133** | **28** | **64** | **72** |

## Table S4: Reliability results by extracted feature

|  |  | **Main analysis (outliers excluded)** | | **Sensitivity analysis (outliers included)** | |
| --- | --- | --- | --- | --- | --- |
| **Challenge** | **Feature** | **ICC (95% CI)** | **median CV (IQR)** | **ICC (95% CI)** | **median CV (IQR)** |
| U-Turn | num_uturns | 0.89 (0.85-0.93) | 0.09 (0.06-0.13) | 0.79 (0.71-0.84) | 0.13 (0.09-0.19) |
|  | mean_uturn_time | 0.36 (0.25-0.45) | 0.07 (0.05-0.13) | 0.24 (0.15-0.33) | 0.11 (0.08-0.20) |
|  | std_uturn_time | 0.26 (0.16-0.35) | 0.50 (0.39-0.70) | 0.08 (0.02-0.15) | 0.83 (0.60-1.21) |
|  | mean_angular_velocity | 0.40 (0.29-0.50) | 0.07 (0.05-0.10) | 0.28 (0.18-0.37) | 0.09 (0.06-0.13) |
|  | std_angular_velocity | 0.22 (0.14-0.32) | 0.48 (0.33-0.67) | 0.04 (0.00-0.09) | 0.66 (0.52-0.87) |
| Two minute walk | num_steps | 0.59 (0.49-0.68) | 0.12 (0.05-0.31) | 0.48 (0.37-0.57) | 0.23 (0.08-0.37) |
|  | mean_step_time | 0.43 (0.32-0.54) | 0.05 (0.03-0.20) | 0.27 (0.17-0.36) | 0.15 (0.04-0.49) |
|  | cadence | 0.45 (0.33-0.55) | 0.05 (0.03-0.25) | 0.34 (0.24-0.44) | 0.12 (0.04-0.32) |
|  | step_time_variability | 0.52 (0.41-0.62) | 0.53 (0.31-0.74) | 0.37 (0.27-0.47) | 0.76 (0.46-1.22) |
|  | step_time_coef_variation | 0.60 (0.50-0.69) | 0.43 (0.23-0.72) | 0.46 (0.35-0.55) | 0.58 (0.36-0.97) |
|  | mean_step_reg | 0.63 (0.52-0.71) | 0.10 (0.03-0.25) | 0.51 (0.40-0.60) | 0.21 (0.07-0.30) |
|  | mean_even_step_reg | 0.57 (0.46-0.66) | 0.11 (0.01-0.25) | 0.49 (0.38-0.60) | 0.17 (0.05-0.29) |
|  | mean_odd_step_reg | 0.59 (0.48-0.68) | 0.09 (0.02-0.27) | 0.50 (0.38-0.59) | 0.20 (0.06-0.29) |
|  | mean_step_dtw | 0.59 (0.47-0.67) | 0.03 (0.01-0.06) | 0.51 (0.39-0.60) | 0.05 (0.02-0.07) |
|  | mean_even_step_dtw | 0.55 (0.44-0.65) | 0.03 (0.01-0.06) | 0.47 (0.36-0.58) | 0.05 (0.02-0.07) |
|  | mean_odd_step_dtw | 0.58 (0.47-0.67) | 0.03 (0.01-0.06) | 0.50 (0.39-0.59) | 0.05 (0.02-0.07) |
|  | mean_step_jerksw | 0.45 (0.34-0.55) | 0.40 (0.19-0.60) | 0.26 (0.17-0.35) | 0.52 (0.31-0.79) |
|  | mean_even_step_jerksw | 0.43 (0.31-0.53) | 0.43 (0.26-0.64) | 0.25 (0.15-0.34) | 0.53 (0.31-0.88) |
|  | mean_odd_step_jerksw | 0.47 (0.34-0.56) | 0.47 (0.29-0.63) | 0.24 (0.15-0.33) | 0.57 (0.38-0.78) |
|  | num_strides | 0.59 (0.49-0.68) | 0.12 (0.05-0.31) | 0.48 (0.37-0.58) | 0.23 (0.07-0.37) |
|  | mean_stride_time | 0.43 (0.32-0.53) | 0.05 (0.03-0.20) | 0.26 (0.18-0.36) | 0.15 (0.04-0.49) |
|  | stride_time_variability | 0.50 (0.39-0.60) | 0.55 (0.29-0.74) | 0.36 (0.25-0.46) | 0.71 (0.49-1.26) |
|  | stride_time_coef_variation | 0.57 (0.47-0.66) | 0.42 (0.23-0.64) | 0.44 (0.33-0.54) | 0.58 (0.43-1.02) |
|  | mean_stride_reg | 0.62 (0.51-0.71) | 0.16 (0.02-0.34) | 0.55 (0.43-0.64) | 0.25 (0.09-0.35) |
|  | mean_stride_dtw | 0.64 (0.53-0.72) | 0.05 (0.02-0.08) | 0.57 (0.46-0.67) | 0.06 (0.04-0.09) |
|  | mean_stride_jerksw | 0.44 (0.32-0.54) | 0.40 (0.19-0.62) | 0.25 (0.16-0.34) | 0.54 (0.31-0.92) |
|  | gait_sym | 0.41 (0.30-0.50) | 0.09 (0.02-0.14) | 0.32 (0.23-0.42) | 0.11 (0.06-0.17) |
|  | jerksw_complete_signal | 0.52 (0.40-0.61) | 0.32 (0.18-0.55) | 0.38 (0.27-0.48) | 0.39 (0.29-0.56) |
|  | num_steps_pedometer | 0.96 (0.94-0.97) | 0.04 (0.03-0.06) | 0.83 (0.76-0.87) | 0.05 (0.03-0.11) |
|  | apple_total_pedometer_dist | 0.61 (0.47-0.71) | 0.08 (0.06-0.11) | 0.40 (0.27-0.52) | 0.11 (0.07-0.15) |
|  | apple_mean_estimated_step_length | 0.51 (0.37-0.62) | 0.06 (0.04-0.11) | 0.44 (0.29-0.56) | 0.09 (0.07-0.12) |
|  | apple_mean_estimated_stride_length | 0.51 (0.37-0.62) | 0.06 (0.04-0.11) | 0.44 (0.30-0.55) | 0.09 (0.07-0.12) |
|  | apple_total_estimated_dist | 0.65 (0.52-0.74) | 0.13 (0.08-0.23) | 0.58 (0.45-0.68) | 0.15 (0.11-0.34) |
| Climbing stairs | num_stairs | 0.86 (0.80-0.89) | 0.00 (0.00-0.11) | 0.75 (0.67-0.81) | 0.10 (0.00-0.20) |
|  | num_steps | 0.68 (0.57-0.75) | 0.14 (0.08-0.27) | 0.28 (0.18-0.37) | 0.19 (0.12-0.56) |
|  | mean_step_time | 0.73 (0.64-0.79) | 0.07 (0.05-0.09) | 0.66 (0.56-0.74) | 0.08 (0.05-0.11) |
|  | cadence | 0.75 (0.66-0.81) | 0.06 (0.04-0.09) | 0.66 (0.56-0.74) | 0.08 (0.05-0.11) |
|  | step_time_variability | 0.72 (0.62-0.78) | 0.38 (0.22-0.56) | 0.65 (0.53-0.72) | 0.41 (0.27-0.63) |
|  | step_time_coef_variation | 0.67 (0.57-0.75) | 0.35 (0.21-0.51) | 0.62 (0.52-0.70) | 0.36 (0.23-0.57) |
|  | mean_step_reg | 0.66 (0.54-0.73) | 0.14 (0.09-0.23) | 0.60 (0.50-0.69) | 0.15 (0.12-0.24) |
|  | mean_even_step_reg | 0.65 (0.53-0.73) | 0.14 (0.09-0.21) | 0.59 (0.49-0.69) | 0.16 (0.11-0.22) |
|  | mean_odd_step_reg | 0.67 (0.56-0.74) | 0.11 (0.07-0.18) | 0.60 (0.49-0.69) | 0.15 (0.10-0.20) |
|  | mean_step_dtw | 0.63 (0.52-0.71) | 0.04 (0.03-0.06) | 0.57 (0.46-0.65) | 0.05 (0.03-0.07) |
|  | mean_even_step_dtw | 0.60 (0.49-0.69) | 0.04 (0.03-0.06) | 0.54 (0.43-0.63) | 0.04 (0.03-0.06) |
|  | mean_odd_step_dtw | 0.66 (0.56-0.74) | 0.03 (0.02-0.05) | 0.58 (0.48-0.67) | 0.04 (0.03-0.05) |
|  | mean_step_jerksw | 0.56 (0.44-0.65) | 0.29 (0.20-0.41) | 0.49 (0.38-0.58) | 0.37 (0.24-0.43) |
|  | mean_even_step_jerksw | 0.56 (0.44-0.65) | 0.34 (0.21-0.44) | 0.46 (0.35-0.55) | 0.39 (0.27-0.46) |
|  | mean_odd_step_jerksw | 0.54 (0.42-0.63) | 0.31 (0.24-0.43) | 0.46 (0.33-0.56) | 0.39 (0.28-0.46) |
|  | num_strides | 0.68 (0.58-0.76) | 0.14 (0.09-0.27) | 0.28 (0.19-0.38) | 0.20 (0.12-0.57) |
|  | mean_stride_time | 0.73 (0.63-0.79) | 0.07 (0.05-0.09) | 0.66 (0.56-0.74) | 0.08 (0.06-0.11) |
|  | stride_time_variability | 0.67 (0.56-0.74) | 0.41 (0.29-0.65) | 0.58 (0.47-0.67) | 0.50 (0.31-0.68) |
|  | stride_time_coef_variation | 0.61 (0.50-0.69) | 0.38 (0.28-0.60) | 0.55 (0.44-0.65) | 0.45 (0.31-0.61) |
|  | mean_stride_reg | 0.65 (0.55-0.73) | 0.17 (0.10-0.23) | 0.58 (0.46-0.67) | 0.19 (0.14-0.26) |
|  | mean_stride_dtw | 0.60 (0.49-0.69) | 0.04 (0.02-0.05) | 0.53 (0.43-0.63) | 0.04 (0.03-0.05) |
|  | mean_stride_jerksw | 0.56 (0.44-0.64) | 0.30 (0.22-0.41) | 0.44 (0.33-0.54) | 0.36 (0.25-0.44) |
|  | gait_sym | 0.25 (0.15-0.33) | 0.10 (0.08-0.13) | 0.18 (0.09-0.26) | 0.11 (0.10-0.15) |
|  | jerksw_complete_signal | 0.55 (0.43-0.64) | 0.26 (0.15-0.37) | 0.28 (0.18-0.38) | 0.30 (0.21-0.47) |
| Musical chairs | num_sitting | 0.58 (0.47-0.67) | 0.16 (0.09-0.23) | 0.50 (0.38-0.59) | 0.19 (0.12-0.29) |
|  | mean_sitting_time | 0.57 (0.47-0.66) | 0.15 (0.07-0.22) | 0.49 (0.38-0.58) | 0.17 (0.11-0.24) |
|  | std_sitting_time | 0.32 (0.22-0.42) | 0.59 (0.42-0.87) | 0.20 (0.11-0.29) | 0.84 (0.50-1.05) |
|  | mean_sitting_velocity | 0.56 (0.44-0.64) | 0.21 (0.15-0.29) | 0.31 (0.21-0.42) | 0.29 (0.19-0.41) |
|  | std_sitting_velocity | 0.26 (0.16-0.35) | 0.54 (0.45-0.72) | 0.02 (0.00-0.07) | 0.67 (0.54-0.89) |
|  | mean_sitting_jerksw | 0.57 (0.45-0.66) | 0.44 (0.33-0.63) | 0.35 (0.24-0.44) | 0.57 (0.41-0.74) |
|  | std_sitting_jerksw | 0.48 (0.36-0.57) | 0.57 (0.46-0.76) | 0.17 (0.09-0.25) | 0.74 (0.53-0.96) |
|  | mean_standing_time | 0.54 (0.42-0.64) | 0.14 (0.07-0.22) | 0.22 (0.13-0.31) | 0.22 (0.12-0.31) |
|  | std_standing_time | 0.36 (0.25-0.46) | 0.59 (0.36-0.85) | 0.06 (0.01-0.12) | 0.90 (0.49-1.28) |
|  | mean_standing_velocity | 0.52 (0.40-0.62) | 0.21 (0.15-0.33) | 0.37 (0.26-0.48) | 0.32 (0.22-0.44) |
|  | std_standing_velocity | 0.28 (0.18-0.38) | 0.54 (0.43-0.72) | 0.07 (0.02-0.14) | 0.68 (0.49-0.86) |
|  | mean_standing_jerksw | 0.57 (0.45-0.66) | 0.48 (0.38-0.61) | 0.36 (0.24-0.46) | 0.59 (0.49-0.81) |
|  | std_standing_jerksw | 0.69 (0.59-0.76) | 0.63 (0.47-0.75) | 0.26 (0.16-0.35) | 0.82 (0.63-1.08) |
|  | mean_complete_move_time | 0.63 (0.51-0.70) | 0.10 (0.07-0.17) | 0.41 (0.30-0.51) | 0.15 (0.11-0.22) |
|  | std_complete_move_time | 0.33 (0.23-0.44) | 0.58 (0.38-0.78) | 0.12 (0.05-0.18) | 0.77 (0.50-1.07) |
| Arm balance | npl_ml (eyes open, right hand) | 0.21 (0.11-0.29) | 0.13 (0.10-0.17) | 0.07 (0.01-0.13) | 0.17 (0.14-0.22) |
|  | npl_ml (eyes closed, right hand) | 0.08 (0.03-0.15) | 0.13 (0.10-0.16) | 0.02 (0.00-0.06) | 0.18 (0.14-0.22) |
|  | npl_ml (eyes open, left hand) | 0.07 (0.01-0.14) | 0.13 (0.08-0.17) | 0.00 (0.00-0.04) | 0.17 (0.13-0.20) |
|  | npl_ml (eyes closed, left hand) | 0.16 (0.08-0.24) | 0.14 (0.10-0.16) | 0.08 (0.02-0.14) | 0.17 (0.15-0.21) |
|  | npl_v (eyes open, right hand) | 0.15 (0.07-0.23) | 0.18 (0.14-0.22) | 0.06 (0.01-0.12) | 0.21 (0.18-0.31) |
|  | npl_v (eyes closed, right hand) | 0.12 (0.06-0.20) | 0.18 (0.14-0.23) | 0.01 (0.00-0.05) | 0.25 (0.19-0.36) |
|  | npl_v (eyes open, left hand) | 0.13 (0.06-0.19) | 0.20 (0.16-0.25) | 0.01 (0.00-0.06) | 0.27 (0.21-0.34) |
|  | npl_v (eyes closed, left hand) | 0.13 (0.05-0.20) | 0.20 (0.14-0.26) | 0.03 (0.00-0.08) | 0.26 (0.19-0.35) |
|  | npl_ap (eyes open, right hand) | 0.29 (0.19-0.39) | 0.01 (0.01-0.01) | 0.11 (0.05-0.18) | 0.01 (0.01-0.02) |
|  | npl_ap (eyes closed, right hand) | 0.80 (0.72-0.85) | 0.01 (0.00-0.01) | 0.57 (0.46-0.67) | 0.01 (0.01-0.01) |
|  | npl_ap (eyes open, left hand) | 0.62 (0.51-0.71) | 0.01 (0.00-0.01) | 0.35 (0.24-0.45) | 0.01 (0.01-0.02) |
|  | npl_ap (eyes closed, left hand) | 0.86 (0.80-0.90) | 0.01 (0.00-0.01) | 0.21 (0.12-0.29) | 0.01 (0.00-0.01) |
|  | mean_velocity_ml (eyes open, right hand) | 0.45 (0.34-0.54) | 0.43 (0.37-0.57) | 0.18 (0.10-0.27) | 0.60 (0.51-0.76) |
|  | mean_velocity_ml (eyes closed, right hand) | 0.46 (0.35-0.55) | 0.41 (0.31-0.51) | 0.27 (0.17-0.37) | 0.56 (0.46-0.63) |
|  | mean_velocity_ml (eyes open, left hand) | 0.40 (0.28-0.50) | 0.48 (0.36-0.58) | 0.19 (0.11-0.27) | 0.54 (0.46-0.63) |
|  | mean_velocity_ml (eyes closed, left hand) | 0.44 (0.33-0.54) | 0.41 (0.33-0.51) | 0.20 (0.11-0.28) | 0.50 (0.41-0.66) |
|  | mean_velocity_v (eyes open, right hand) | 0.43 (0.34-0.54) | 0.53 (0.41-0.68) | 0.10 (0.03-0.17) | 0.69 (0.60-0.87) |
|  | mean_velocity_v (eyes closed, right hand) | 0.40 (0.29-0.50) | 0.50 (0.42-0.59) | 0.24 (0.14-0.33) | 0.57 (0.48-0.71) |
|  | mean_velocity_v (eyes open, left hand) | 0.28 (0.18-0.38) | 0.53 (0.44-0.65) | 0.09 (0.03-0.15) | 0.64 (0.56-0.78) |
|  | mean_velocity_v (eyes closed, left hand) | 0.40 (0.29-0.49) | 0.55 (0.42-0.60) | 0.17 (0.09-0.25) | 0.59 (0.50-0.66) |
|  | mean_velocity_ap (eyes open, right hand) | 0.30 (0.20-0.40) | 0.03 (0.02-0.05) | 0.11 (0.04-0.17) | 0.04 (0.03-0.07) |
|  | mean_velocity_ap (eyes closed, right hand) | 0.46 (0.35-0.57) | 0.03 (0.02-0.04) | 0.29 (0.19-0.38) | 0.03 (0.02-0.04) |
|  | mean_velocity_ap (eyes open, left hand) | 0.22 (0.13-0.30) | 0.03 (0.01-0.04) | 0.07 (0.02-0.14) | 0.03 (0.02-0.05) |
|  | mean_velocity_ap (eyes closed, left hand) | 0.41 (0.31-0.52) | 0.02 (0.01-0.03) | 0.14 (0.07-0.22) | 0.03 (0.02-0.03) |
|  | rms_tremor_gyr (eyes open, right hand) | 0.52 (0.39-0.61) | 0.31 (0.24-0.38) | 0.30 (0.20-0.40) | 0.38 (0.31-0.47) |
|  | rms_tremor_gyr (eyes closed, right hand) | 0.53 (0.42-0.62) | 0.26 (0.20-0.35) | 0.29 (0.19-0.39) | 0.33 (0.25-0.38) |
|  | rms_tremor_gyr (eyes open, left hand) | 0.50 (0.39-0.60) | 0.29 (0.21-0.39) | 0.40 (0.29-0.50) | 0.34 (0.27-0.42) |
|  | rms_tremor_gyr (eyes closed, left hand) | 0.48 (0.37-0.58) | 0.26 (0.19-0.39) | 0.25 (0.16-0.35) | 0.29 (0.21-0.40) |
|  | total_power_gyr (eyes open, right hand) | 0.50 (0.38-0.59) | 0.32 (0.25-0.42) | 0.29 (0.18-0.38) | 0.41 (0.30-0.54) |
|  | total_power_gyr (eyes closed, right hand) | 0.47 (0.35-0.57) | 0.28 (0.19-0.34) | 0.23 (0.14-0.32) | 0.33 (0.26-0.42) |
|  | total_power_gyr (eyes open, left hand) | 0.51 (0.39-0.60) | 0.29 (0.20-0.39) | 0.41 (0.30-0.52) | 0.35 (0.28-0.48) |
|  | total_power_gyr (eyes closed, left hand) | 0.49 (0.38-0.59) | 0.28 (0.18-0.37) | 0.20 (0.12-0.29) | 0.31 (0.25-0.38) |
|  | peak_power_gyr (eyes open, right hand) | 0.47 (0.35-0.57) | 0.35 (0.29-0.42) | 0.24 (0.15-0.33) | 0.42 (0.31-0.51) |
|  | peak_power_gyr (eyes closed, right hand) | 0.45 (0.33-0.56) | 0.30 (0.23-0.42) | 0.20 (0.11-0.28) | 0.37 (0.28-0.46) |
|  | peak_power_gyr (eyes open, left hand) | 0.46 (0.35-0.56) | 0.32 (0.26-0.40) | 0.31 (0.20-0.40) | 0.38 (0.31-0.45) |
|  | peak_power_gyr (eyes closed, left hand) | 0.47 (0.35-0.57) | 0.32 (0.25-0.38) | 0.21 (0.13-0.30) | 0.35 (0.28-0.44) |
|  | rms_tremor_acc (eyes open, right hand) | 0.66 (0.56-0.74) | 0.25 (0.21-0.32) | 0.42 (0.30-0.51) | 0.32 (0.25-0.42) |
|  | rms_tremor_acc (eyes closed, right hand) | 0.61 (0.51-0.70) | 0.23 (0.13-0.30) | 0.51 (0.41-0.61) | 0.24 (0.22-0.31) |
|  | rms_tremor_acc (eyes open, left hand) | 0.72 (0.63-0.79) | 0.23 (0.16-0.30) | 0.64 (0.54-0.71) | 0.27 (0.21-0.34) |
|  | rms_tremor_acc (eyes closed, left hand) | 0.63 (0.52-0.71) | 0.21 (0.16-0.27) | 0.44 (0.33-0.54) | 0.23 (0.19-0.32) |
|  | total_power_acc (eyes open, right hand) | 0.61 (0.51-0.69) | 0.31 (0.24-0.36) | 0.40 (0.28-0.49) | 0.36 (0.28-0.49) |
|  | total_power_acc (eyes closed, right hand) | 0.54 (0.43-0.63) | 0.26 (0.21-0.33) | 0.44 (0.33-0.54) | 0.31 (0.25-0.39) |
|  | total_power_acc (eyes open, left hand) | 0.64 (0.54-0.72) | 0.29 (0.21-0.35) | 0.60 (0.49-0.68) | 0.31 (0.25-0.37) |
|  | total_power_acc (eyes closed, left hand) | 0.64 (0.53-0.71) | 0.26 (0.22-0.35) | 0.36 (0.25-0.46) | 0.27 (0.22-0.37) |
|  | peak_power_acc (eyes open, right hand) | 0.51 (0.40-0.61) | 0.34 (0.25-0.39) | 0.32 (0.23-0.42) | 0.42 (0.34-0.55) |
|  | peak_power_acc (eyes closed, right hand) | 0.47 (0.35-0.57) | 0.33 (0.25-0.40) | 0.35 (0.24-0.45) | 0.38 (0.32-0.47) |
|  | peak_power_acc (eyes open, left hand) | 0.59 (0.48-0.68) | 0.29 (0.21-0.40) | 0.52 (0.41-0.61) | 0.36 (0.29-0.43) |
|  | peak_power_acc (eyes closed, left hand) | 0.50 (0.39-0.60) | 0.30 (0.24-0.41) | 0.39 (0.27-0.49) | 0.35 (0.28-0.45) |
| Standing balance | npl_ml (eyes open) | 0.03 (0.00-0.09) | 0.16 (0.12-0.19) | 0.01 (0.00-0.05) | 0.18 (0.15-0.21) |
|  | npl_ml (eyes closed) | 0.13 (0.06-0.21) | 0.15 (0.11-0.19) | 0.06 (0.01-0.13) | 0.17 (0.14-0.20) |
|  | npl_ap (eyes open) | 0.81 (0.73-0.86) | 0.00 (0.00-0.01) | 0.17 (0.09-0.24) | 0.01 (0.00-0.01) |
|  | npl_ap (eyes closed) | 0.93 (0.90-0.95) | 0.00 (0.00-0.00) | 0.34 (0.24-0.44) | 0.00 (0.00-0.01) |
|  | jerksw (eyes open) | 0.40 (0.29-0.49) | 0.66 (0.49-0.80) | 0.00 (0.00-0.04) | 1.10 (0.75-1.64) |
|  | jerksw (eyes closed) | 0.48 (0.36-0.57) | 0.58 (0.43-0.67) | 0.00 (0.00-0.05) | 0.75 (0.59-1.28) |
| Screen-to-nose | num_green_touches (eyes open, right hand) | 0.65 (0.54-0.73) | 0.17 (0.10-0.27) | 0.33 (0.23-0.43) | 0.22 (0.15-0.30) |
|  | num_green_touches (eyes closed, right hand) | 0.63 (0.53-0.71) | 0.30 (0.16-0.47) | 0.37 (0.26-0.47) | 0.34 (0.23-0.50) |
|  | num_green_touches (eyes open, left hand) | 0.65 (0.55-0.73) | 0.15 (0.10-0.23) | 0.35 (0.24-0.45) | 0.19 (0.13-0.26) |
|  | num_green_touches (eyes closed, left hand) | 0.68 (0.58-0.75) | 0.21 (0.14-0.40) | 0.43 (0.32-0.53) | 0.28 (0.19-0.41) |
|  | num_yellow_touches (eyes open, right hand) | 0.46 (0.34-0.55) | 1.06 (0.86-1.56) | 0.32 (0.22-0.41) | 1.61 (0.97-2.25) |
|  | num_yellow_touches (eyes closed, right hand) | 0.41 (0.31-0.51) | 0.94 (0.80-1.27) | 0.33 (0.23-0.42) | 1.09 (0.84-1.55) |
|  | num_yellow_touches (eyes open, left hand) | 0.49 (0.37-0.58) | 1.19 (0.86-1.50) | 0.37 (0.26-0.46) | 1.67 (1.10-3.16) |
|  | num_yellow_touches (eyes closed, left hand) | 0.41 (0.29-0.51) | 0.95 (0.75-1.26) | 0.17 (0.09-0.25) | 1.10 (0.91-1.42) |
|  | num_total_touches (eyes open, right hand) | 0.70 (0.60-0.77) | 0.12 (0.08-0.22) | 0.36 (0.25-0.46) | 0.19 (0.11-0.25) |
|  | num_total_touches (eyes closed, right hand) | 0.67 (0.57-0.75) | 0.12 (0.08-0.17) | 0.38 (0.28-0.48) | 0.18 (0.13-0.23) |
|  | num_total_touches (eyes open, left hand) | 0.70 (0.60-0.78) | 0.11 (0.08-0.17) | 0.37 (0.27-0.47) | 0.15 (0.11-0.21) |
|  | num_total_touches (eyes closed, left hand) | 0.72 (0.63-0.79) | 0.12 (0.07-0.16) | 0.39 (0.28-0.49) | 0.15 (0.11-0.19) |
|  | mean_mm_distance (eyes open, right hand) | 0.53 (0.42-0.62) | 0.23 (0.16-0.29) | 0.32 (0.21-0.41) | 0.29 (0.21-0.38) |
|  | mean_mm_distance (eyes closed, right hand) | 0.45 (0.34-0.55) | 0.27 (0.23-0.35) | 0.37 (0.27-0.47) | 0.30 (0.26-0.37) |
|  | mean_mm_distance (eyes open, left hand) | 0.58 (0.47-0.66) | 0.20 (0.16-0.28) | 0.29 (0.19-0.38) | 0.26 (0.20-0.35) |
|  | mean_mm_distance (eyes closed, left hand) | 0.44 (0.32-0.54) | 0.26 (0.19-0.34) | 0.40 (0.29-0.49) | 0.29 (0.24-0.35) |
|  | std_mm_distance (eyes open, right hand) | 0.36 (0.25-0.46) | 0.28 (0.22-0.35) | 0.12 (0.05-0.19) | 0.36 (0.27-0.53) |
|  | std_mm_distance (eyes closed, right hand) | 0.33 (0.22-0.43) | 0.29 (0.22-0.39) | 0.26 (0.16-0.35) | 0.34 (0.29-0.41) |
|  | std_mm_distance (eyes open, left hand) | 0.36 (0.25-0.45) | 0.26 (0.19-0.36) | 0.19 (0.10-0.27) | 0.35 (0.25-0.51) |
|  | std_mm_distance (eyes closed, left hand) | 0.35 (0.24-0.45) | 0.28 (0.20-0.33) | 0.24 (0.15-0.33) | 0.32 (0.26-0.43) |
|  | num_bends (eyes open, right hand) | 0.60 (0.48-0.68) | 0.13 (0.08-0.17) | 0.54 (0.42-0.63) | 0.14 (0.10-0.19) |
|  | num_bends (eyes closed, right hand) | 0.67 (0.57-0.74) | 0.12 (0.08-0.16) | 0.60 (0.48-0.68) | 0.15 (0.11-0.19) |
|  | num_bends (eyes open, left hand) | 0.59 (0.48-0.67) | 0.13 (0.10-0.17) | 0.54 (0.42-0.64) | 0.14 (0.11-0.19) |
|  | num_bends (eyes closed, left hand) | 0.66 (0.56-0.74) | 0.13 (0.08-0.17) | 0.61 (0.51-0.70) | 0.14 (0.12-0.18) |
|  | mean_bend_time (eyes open, right hand) | 0.55 (0.43-0.64) | 0.18 (0.14-0.27) | 0.42 (0.31-0.52) | 0.25 (0.19-0.34) |
|  | mean_bend_time (eyes closed, right hand) | 0.66 (0.55-0.74) | 0.17 (0.12-0.25) | 0.52 (0.40-0.61) | 0.25 (0.17-0.32) |
|  | mean_bend_time (eyes open, left hand) | 0.55 (0.44-0.64) | 0.19 (0.15-0.27) | 0.51 (0.40-0.61) | 0.23 (0.18-0.30) |
|  | mean_bend_time (eyes closed, left hand) | 0.63 (0.52-0.70) | 0.17 (0.10-0.26) | 0.57 (0.47-0.65) | 0.21 (0.16-0.28) |
|  | std_bend_time (eyes open, right hand) | 0.31 (0.21-0.41) | 0.52 (0.41-0.77) | 0.22 (0.14-0.31) | 0.64 (0.47-0.83) |
|  | std_bend_time (eyes closed, right hand) | 0.34 (0.24-0.45) | 0.53 (0.40-0.73) | 0.25 (0.15-0.34) | 0.69 (0.49-0.92) |
|  | std_bend_time (eyes open, left hand) | 0.34 (0.23-0.44) | 0.54 (0.43-0.65) | 0.26 (0.17-0.35) | 0.60 (0.51-0.76) |
|  | std_bend_time (eyes closed, left hand) | 0.35 (0.24-0.45) | 0.50 (0.41-0.69) | 0.30 (0.20-0.39) | 0.57 (0.48-0.81) |
|  | mean_stretch_time (eyes open, right hand) | 0.61 (0.50-0.69) | 0.18 (0.11-0.24) | 0.55 (0.43-0.64) | 0.22 (0.17-0.27) |
|  | mean_stretch_time (eyes closed, right hand) | 0.64 (0.53-0.71) | 0.17 (0.12-0.23) | 0.56 (0.44-0.65) | 0.21 (0.15-0.25) |
|  | mean_stretch_time (eyes open, left hand) | 0.53 (0.42-0.62) | 0.17 (0.12-0.25) | 0.43 (0.33-0.53) | 0.22 (0.16-0.31) |
|  | mean_stretch_time (eyes closed, left hand) | 0.59 (0.48-0.68) | 0.18 (0.10-0.25) | 0.50 (0.38-0.59) | 0.22 (0.15-0.30) |
|  | std_stretch_time (eyes open, right hand) | 0.42 (0.31-0.50) | 0.54 (0.41-0.67) | 0.33 (0.22-0.42) | 0.61 (0.47-0.78) |
|  | std_stretch_time (eyes closed, right hand) | 0.45 (0.35-0.55) | 0.56 (0.40-0.70) | 0.35 (0.25-0.45) | 0.66 (0.47-0.82) |
|  | std_stretch_time (eyes open, left hand) | 0.41 (0.31-0.51) | 0.61 (0.45-0.71) | 0.26 (0.17-0.36) | 0.67 (0.50-0.84) |
|  | std_stretch_time (eyes closed, left hand) | 0.36 (0.24-0.46) | 0.54 (0.43-0.59) | 0.26 (0.17-0.36) | 0.63 (0.49-0.77) |
|  | mean_bend_jerksw (eyes open, right hand) | 0.63 (0.53-0.71) | 0.35 (0.26-0.48) | 0.61 (0.49-0.69) | 0.46 (0.35-0.61) |
|  | mean_bend_jerksw (eyes closed, right hand) | 0.72 (0.64-0.79) | 0.39 (0.29-0.49) | 0.68 (0.58-0.75) | 0.44 (0.35-0.54) |
|  | mean_bend_jerksw (eyes open, left hand) | 0.80 (0.72-0.85) | 0.32 (0.23-0.41) | 0.68 (0.58-0.75) | 0.35 (0.29-0.53) |
|  | mean_bend_jerksw (eyes closed, left hand) | 0.75 (0.65-0.80) | 0.33 (0.24-0.41) | 0.68 (0.58-0.76) | 0.36 (0.28-0.48) |
|  | std_bend_jerksw (eyes open, right hand) | 0.59 (0.47-0.68) | 0.49 (0.41-0.65) | 0.47 (0.36-0.56) | 0.61 (0.48-0.73) |
|  | std_bend_jerksw (eyes closed, right hand) | 0.56 (0.44-0.65) | 0.56 (0.46-0.68) | 0.47 (0.36-0.57) | 0.65 (0.49-0.77) |
|  | std_bend_jerksw (eyes open, left hand) | 0.57 (0.46-0.66) | 0.45 (0.40-0.60) | 0.50 (0.39-0.60) | 0.53 (0.42-0.72) |
|  | std_bend_jerksw (eyes closed, left hand) | 0.58 (0.47-0.67) | 0.49 (0.38-0.61) | 0.45 (0.34-0.55) | 0.54 (0.44-0.72) |
|  | mean_stretch_jerksw (eyes open, right hand) | 0.72 (0.63-0.79) | 0.39 (0.27-0.51) | 0.67 (0.57-0.74) | 0.43 (0.29-0.58) |
|  | mean_stretch_jerksw (eyes closed, right hand) | 0.68 (0.57-0.75) | 0.38 (0.25-0.47) | 0.66 (0.54-0.74) | 0.40 (0.29-0.55) |
|  | mean_stretch_jerksw (eyes open, left hand) | 0.72 (0.63-0.78) | 0.37 (0.23-0.47) | 0.65 (0.54-0.73) | 0.43 (0.31-0.54) |
|  | mean_stretch_jerksw (eyes closed, left hand) | 0.75 (0.66-0.81) | 0.36 (0.25-0.47) | 0.71 (0.62-0.78) | 0.38 (0.31-0.49) |
|  | std_stretch_jerksw (eyes open, right hand) | 0.71 (0.61-0.78) | 0.51 (0.44-0.64) | 0.54 (0.42-0.63) | 0.62 (0.51-0.79) |
|  | std_stretch_jerksw (eyes closed, right hand) | 0.57 (0.46-0.66) | 0.51 (0.42-0.64) | 0.52 (0.40-0.62) | 0.64 (0.48-0.81) |
|  | std_stretch_jerksw (eyes open, left hand) | 0.60 (0.48-0.68) | 0.47 (0.37-0.61) | 0.44 (0.33-0.54) | 0.59 (0.46-0.76) |
|  | std_stretch_jerksw (eyes closed, left hand) | 0.55 (0.44-0.64) | 0.52 (0.41-0.64) | 0.54 (0.44-0.63) | 0.56 (0.47-0.75) |
|  | mean_bend_rms_tremor_acc (eyes open, right hand) | 0.84 (0.77-0.88) | 0.19 (0.15-0.28) | 0.80 (0.72-0.85) | 0.23 (0.18-0.31) |
|  | mean_bend_rms_tremor_acc (eyes closed, right hand) | 0.84 (0.77-0.88) | 0.17 (0.12-0.27) | 0.80 (0.73-0.85) | 0.23 (0.17-0.31) |
|  | mean_bend_rms_tremor_acc (eyes open, left hand) | 0.85 (0.79-0.89) | 0.16 (0.13-0.22) | 0.82 (0.75-0.87) | 0.19 (0.15-0.24) |
|  | mean_bend_rms_tremor_acc (eyes closed, left hand) | 0.85 (0.79-0.89) | 0.16 (0.13-0.22) | 0.82 (0.76-0.87) | 0.19 (0.15-0.25) |
|  | std_bend_rms_tremor_acc (eyes open, right hand) | 0.53 (0.42-0.62) | 0.44 (0.35-0.51) | 0.47 (0.36-0.56) | 0.47 (0.43-0.54) |
|  | std_bend_rms_tremor_acc (eyes closed, right hand) | 0.60 (0.49-0.68) | 0.42 (0.36-0.51) | 0.48 (0.36-0.56) | 0.49 (0.41-0.56) |
|  | std_bend_rms_tremor_acc (eyes open, left hand) | 0.54 (0.43-0.63) | 0.42 (0.33-0.51) | 0.51 (0.39-0.61) | 0.44 (0.36-0.55) |
|  | std_bend_rms_tremor_acc (eyes closed, left hand) | 0.59 (0.48-0.68) | 0.40 (0.33-0.52) | 0.53 (0.42-0.62) | 0.46 (0.37-0.60) |
|  | mean_stretch_rms_tremor_acc (eyes open, right hand) | 0.81 (0.74-0.86) | 0.20 (0.13-0.26) | 0.80 (0.72-0.85) | 0.22 (0.16-0.28) |
|  | mean_stretch_rms_tremor_acc (eyes closed, right hand) | 0.85 (0.80-0.89) | 0.18 (0.11-0.24) | 0.79 (0.72-0.85) | 0.22 (0.18-0.28) |
|  | mean_stretch_rms_tremor_acc (eyes open, left hand) | 0.83 (0.77-0.88) | 0.17 (0.13-0.26) | 0.81 (0.74-0.86) | 0.21 (0.16-0.27) |
|  | mean_stretch_rms_tremor_acc (eyes closed, left hand) | 0.82 (0.75-0.87) | 0.18 (0.15-0.27) | 0.80 (0.72-0.85) | 0.21 (0.17-0.28) |
|  | std_stretch_rms_tremor_acc (eyes open, right hand) | 0.55 (0.43-0.63) | 0.45 (0.38-0.55) | 0.46 (0.35-0.56) | 0.49 (0.41-0.58) |
|  | std_stretch_rms_tremor_acc (eyes closed, right hand) | 0.59 (0.48-0.67) | 0.42 (0.30-0.55) | 0.50 (0.39-0.59) | 0.51 (0.38-0.58) |
|  | std_stretch_rms_tremor_acc (eyes open, left hand) | 0.52 (0.41-0.61) | 0.42 (0.36-0.51) | 0.47 (0.36-0.56) | 0.48 (0.37-0.59) |
|  | std_stretch_rms_tremor_acc (eyes closed, left hand) | 0.55 (0.44-0.64) | 0.44 (0.36-0.55) | 0.50 (0.39-0.59) | 0.48 (0.38-0.57) |
|  | mean_bend_rms_tremor_gyr (eyes open, right hand) | 0.83 (0.77-0.88) | 0.18 (0.14-0.26) | 0.78 (0.70-0.83) | 0.22 (0.17-0.29) |
|  | mean_bend_rms_tremor_gyr (eyes closed, right hand) | 0.80 (0.73-0.85) | 0.17 (0.13-0.26) | 0.76 (0.67-0.82) | 0.21 (0.17-0.27) |
|  | mean_bend_rms_tremor_gyr (eyes open, left hand) | 0.80 (0.72-0.85) | 0.14 (0.11-0.19) | 0.74 (0.65-0.80) | 0.17 (0.13-0.22) |
|  | mean_bend_rms_tremor_gyr (eyes closed, left hand) | 0.78 (0.69-0.83) | 0.16 (0.12-0.21) | 0.72 (0.63-0.79) | 0.17 (0.13-0.22) |
|  | std_bend_rms_tremor_gyr (eyes open, right hand) | 0.41 (0.30-0.50) | 0.40 (0.34-0.49) | 0.37 (0.27-0.47) | 0.43 (0.36-0.51) |
|  | std_bend_rms_tremor_gyr (eyes closed, right hand) | 0.49 (0.37-0.58) | 0.42 (0.37-0.49) | 0.45 (0.34-0.55) | 0.43 (0.40-0.56) |
|  | std_bend_rms_tremor_gyr (eyes open, left hand) | 0.40 (0.29-0.49) | 0.39 (0.35-0.49) | 0.35 (0.25-0.44) | 0.43 (0.37-0.51) |
|  | std_bend_rms_tremor_gyr (eyes closed, left hand) | 0.46 (0.35-0.56) | 0.41 (0.31-0.51) | 0.40 (0.29-0.49) | 0.47 (0.36-0.55) |
|  | mean_stretch_rms_tremor_gyr (eyes open, right hand) | 0.79 (0.71-0.84) | 0.16 (0.11-0.22) | 0.74 (0.64-0.80) | 0.19 (0.14-0.26) |
|  | mean_stretch_rms_tremor_gyr (eyes closed, right hand) | 0.78 (0.71-0.84) | 0.16 (0.12-0.21) | 0.73 (0.64-0.80) | 0.20 (0.16-0.26) |
|  | mean_stretch_rms_tremor_gyr (eyes open, left hand) | 0.80 (0.73-0.86) | 0.17 (0.13-0.22) | 0.75 (0.66-0.81) | 0.20 (0.16-0.25) |
|  | mean_stretch_rms_tremor_gyr (eyes closed, left hand) | 0.76 (0.68-0.82) | 0.18 (0.14-0.24) | 0.73 (0.63-0.79) | 0.20 (0.17-0.26) |
|  | std_stretch_rms_tremor_gyr (eyes open, right hand) | 0.48 (0.37-0.58) | 0.42 (0.33-0.48) | 0.38 (0.27-0.48) | 0.45 (0.38-0.51) |
|  | std_stretch_rms_tremor_gyr (eyes closed, right hand) | 0.53 (0.41-0.62) | 0.39 (0.30-0.47) | 0.45 (0.34-0.55) | 0.43 (0.38-0.55) |
|  | std_stretch_rms_tremor_gyr (eyes open, left hand) | 0.45 (0.33-0.54) | 0.39 (0.29-0.47) | 0.37 (0.25-0.47) | 0.42 (0.36-0.52) |
|  | std_stretch_rms_tremor_gyr (eyes closed, left hand) | 0.46 (0.35-0.56) | 0.44 (0.34-0.52) | 0.40 (0.30-0.48) | 0.46 (0.37-0.59) |
|  | mean_bend_velocity (eyes open, right hand) | 0.69 (0.59-0.76) | 0.22 (0.15-0.32) | 0.59 (0.48-0.68) | 0.24 (0.20-0.34) |
|  | mean_bend_velocity (eyes closed, right hand) | 0.68 (0.58-0.75) | 0.20 (0.15-0.29) | 0.62 (0.51-0.71) | 0.24 (0.18-0.34) |
|  | mean_bend_velocity (eyes open, left hand) | 0.63 (0.52-0.71) | 0.23 (0.16-0.32) | 0.59 (0.49-0.68) | 0.28 (0.20-0.36) |
|  | mean_bend_velocity (eyes closed, left hand) | 0.64 (0.54-0.72) | 0.24 (0.16-0.30) | 0.59 (0.49-0.68) | 0.25 (0.20-0.35) |
|  | std_bend_velocity (eyes open, right hand) | 0.36 (0.25-0.46) | 0.54 (0.43-0.61) | 0.26 (0.17-0.35) | 0.60 (0.52-0.74) |
|  | std_bend_velocity (eyes closed, right hand) | 0.61 (0.50-0.69) | 0.50 (0.38-0.64) | 0.40 (0.29-0.48) | 0.63 (0.51-0.76) |
|  | std_bend_velocity (eyes open, left hand) | 0.48 (0.38-0.58) | 0.46 (0.37-0.57) | 0.32 (0.21-0.42) | 0.59 (0.45-0.71) |
|  | std_bend_velocity (eyes closed, left hand) | 0.36 (0.25-0.46) | 0.54 (0.48-0.69) | 0.26 (0.16-0.35) | 0.62 (0.50-0.74) |
|  | mean_stretch_velocity (eyes open, right hand) | 0.65 (0.56-0.73) | 0.21 (0.16-0.28) | 0.58 (0.48-0.67) | 0.25 (0.20-0.33) |
|  | mean_stretch_velocity (eyes closed, right hand) | 0.71 (0.61-0.77) | 0.22 (0.18-0.29) | 0.62 (0.50-0.70) | 0.23 (0.20-0.36) |
|  | mean_stretch_velocity (eyes open, left hand) | 0.66 (0.55-0.74) | 0.21 (0.14-0.27) | 0.61 (0.51-0.69) | 0.26 (0.21-0.36) |
|  | mean_stretch_velocity (eyes closed, left hand) | 0.65 (0.54-0.73) | 0.21 (0.16-0.32) | 0.62 (0.52-0.70) | 0.24 (0.19-0.36) |
|  | std_stretch_velocity (eyes open, right hand) | 0.36 (0.25-0.45) | 0.53 (0.45-0.64) | 0.24 (0.14-0.32) | 0.64 (0.50-0.72) |
|  | std_stretch_velocity (eyes closed, right hand) | 0.42 (0.31-0.52) | 0.55 (0.41-0.68) | 0.27 (0.18-0.35) | 0.63 (0.45-0.80) |
|  | std_stretch_velocity (eyes open, left hand) | 0.51 (0.40-0.61) | 0.47 (0.40-0.57) | 0.41 (0.30-0.51) | 0.57 (0.47-0.72) |
|  | std_stretch_velocity (eyes closed, left hand) | 0.45 (0.34-0.55) | 0.50 (0.44-0.59) | 0.34 (0.25-0.44) | 0.58 (0.49-0.69) |
|  | mean_full_movement_time (eyes open, right hand) | 0.66 (0.56-0.73) | 0.13 (0.09-0.20) | 0.62 (0.51-0.70) | 0.16 (0.11-0.20) |
|  | mean_full_movement_time (eyes closed, right hand) | 0.73 (0.64-0.80) | 0.13 (0.09-0.16) | 0.65 (0.55-0.73) | 0.16 (0.12-0.20) |
|  | mean_full_movement_time (eyes open, left hand) | 0.63 (0.52-0.72) | 0.14 (0.09-0.18) | 0.58 (0.47-0.67) | 0.15 (0.11-0.20) |
|  | mean_full_movement_time (eyes closed, left hand) | 0.70 (0.61-0.78) | 0.13 (0.10-0.17) | 0.63 (0.51-0.71) | 0.16 (0.11-0.20) |
|  | std_full_movement_time (eyes open, right hand) | 0.44 (0.33-0.54) | 0.47 (0.37-0.67) | 0.33 (0.23-0.43) | 0.62 (0.44-0.82) |
|  | std_full_movement_time (eyes closed, right hand) | 0.43 (0.32-0.53) | 0.50 (0.36-0.67) | 0.34 (0.24-0.43) | 0.69 (0.45-0.86) |
|  | std_full_movement_time (eyes open, left hand) | 0.44 (0.33-0.54) | 0.48 (0.40-0.70) | 0.34 (0.24-0.44) | 0.62 (0.44-0.81) |
|  | std_full_movement_time (eyes closed, left hand) | 0.43 (0.32-0.53) | 0.47 (0.35-0.59) | 0.37 (0.27-0.46) | 0.52 (0.44-0.74) |
| Catch-a-cloud | num_successful_touches | 0.92 (0.89-0.94) | 0.05 (0.03-0.06) | 0.89 (0.84-0.92) | 0.06 (0.04-0.08) |
|  | num_total_touches | 0.93 (0.90-0.95) | 0.05 (0.04-0.05) | 0.90 (0.85-0.92) | 0.06 (0.04-0.07) |
|  | mean_mm_distance | 0.50 (0.37-0.62) | 0.11 (0.08-0.13) | 0.26 (0.15-0.38) | 0.12 (0.10-0.18) |
|  | std_mm_distance | 0.26 (0.15-0.36) | 0.16 (0.13-0.19) | 0.10 (0.02-0.18) | 0.31 (0.16-0.74) |
| m-SDMT | correct_responses_trial_1 | 0.85 (0.78-0.90) | 0.00 (0.00-0.00) | 0.52 (0.39-0.62) | 0.00 (0.00-0.06) |
|  | correct_responses_trial_2 | 0.56 (0.43-0.66) | 0.00 (0.00-0.00) | 0.36 (0.23-0.47) | 0.05 (0.00-0.45) |
|  | correct_responses_trial_3 | 0.70 (0.59-0.78) | 0.00 (0.00-0.00) | 0.47 (0.34-0.58) | 0.00 (0.00-0.06) |
|  | correct_responses_trial_4 | 0.72 (0.61-0.79) | 0.00 (0.00-0.00) | 0.46 (0.33-0.57) | 0.05 (0.00-0.10) |
|  | correct_responses_trial_5 | 0.69 (0.59-0.77) | 0.00 (0.00-0.00) | 0.48 (0.34-0.58) | 0.00 (0.00-0.05) |
|  | correct_responses_trial_6 | 0.64 (0.52-0.73) | 0.00 (0.00-0.00) | 0.47 (0.34-0.58) | 0.00 (0.00-0.06) |
|  | correct_responses_trial_7 | 0.65 (0.53-0.73) | 0.00 (0.00-0.00) | 0.46 (0.33-0.57) | 0.00 (0.00-0.05) |
|  | correct_responses_trial_8 | 0.69 (0.58-0.77) | 0.00 (0.00-0.00) | 0.46 (0.33-0.57) | 0.00 (0.00-0.08) |
|  | total_correct_responses | 0.68 (0.57-0.76) | 0.01 (0.00-0.04) | 0.60 (0.48-0.70) | 0.04 (0.01-0.08) |
|  | mean_time_response | 0.76 (0.67-0.83) | 0.06 (0.03-0.09) | 0.75 (0.65-0.82) | 0.08 (0.06-0.11) |
| Near vision | final_score_left_eye | 0.85 (0.77-0.89) | 0.12 (0.00-0.19) | 0.80 (0.71-0.85) | 0.16 (0.12-0.20) |
|  | final_score_right_eye | 0.77 (0.68-0.83) | 0.13 (0.00-0.20) | 0.72 (0.61-0.79) | 0.19 (0.12-0.25) |
| Low contrast | final_score_left_eye | 0.89 (0.84-0.93) | 0.00 (0.00-0.04) | 0.87 (0.82-0.91) | 0.04 (0.03-0.06) |
|  | final_score_right_eye | 0.81 (0.73-0.86) | 0.00 (0.00-0.05) | 0.66 (0.55-0.75) | 0.04 (0.03-0.06) |

ICC ≥ 0.6 and mCV < 0.2 highlighted in green.

## Table S5: Acceptance questionnaire

| **Topic** | **German version** | **English version** |
| --- | --- | --- |
| **Overall impression** | Hat Ihnen diese Übung grundsätzlich gut gefallen? | Did you like this exercise in principle? |
| **Perceived difficulty** | Fühlten Sie sich bei der Durchführung dieser Übung positiv herausgefordert (sie war nicht zu einfach aber auch nicht zu schwierig)? | Did you feel positively challenged in carrying out this exercise (it was neither too easy nor too difficult)? |
| **Willingness of future use** | Wären Sie bereit, diese Übung auch in Zukunft regelmässig durchzuführen? | Would you be willing to regularly carry out this exercise in the future? |
| **MS relevance (only PwMS)** | Meinen Sie, dass diese Übung sinnvoll ist für Menschen mit Multipler Sklerose? | Do you think this exercise makes sense for people with multiple sclerosis? |

## Table S6: Smartwatch median daily features in HV and PwMS

|  | **Healthy volunteers (n=27)** | **People with MS (n=28)** | **Rank-biserial r (95% CI)** |
| --- | --- | --- | --- |
| **Activity features** | | | |
| Steps | **10338 (IQR 8358.2-11294, range 2770-16783.5)** | **7188 (IQR 5182.2-8900.8, range 871-13962)** | **0.51 (0.24 to 0.70)** |
| Distance (km) | **7.3 (IQR 5.7-8.2, range 1.9-12.1)** | **5.3 (IQR 3.5-6.2, range 0.6-9.4)** | **0.48 (0.21 to 0.68)** |
| Floors | 10 (IQR 5.5-18, range 1-36) | 7.2 (IQR 3-13.4, range 0-25.5) | 0.25 (-0.05 to 0.51) |
| Calories (kcal) | 2254.7 (IQR 2104.3-2986.3, range 1841.4-3683) | 2258.2 (IQR 1990.6-2681.6, range 1462.8-3157.9) | 0.13 (-0.17 to 0.42) |
| Calories (MET) | **23128 (IQR 22359.8-25418, range 16635.5-30391.5)** | **21632.2 (IQR 20088.5-24102.8, range 16397-28316.5)** | **0.31 (0.02 to 0.56)** |
| Proportion sedentary | 0.8 (IQR 0.8-0.8, range 0.7-0.9) | 0.8 (IQR 0.7-0.9, range 0.6-1) | -0.19 (-0.46 to 0.12) |
| Proportion lightly active | 0.2 (IQR 0.2-0.2, range 0.1-0.3) | 0.2 (IQR 0.1-0.2, range 0-0.3) | 0.08 (-0.22 to 0.37) |
| Proportion fairly active | **0.008 (IQR 0.0043-0.0149, range 0-0.0299)** | **0.0033 (IQR 0-0.0099, range 0-0.0295)** | **0.37 (0.08 to 0.60)** |
| Proportion very active | **0.0111 (IQR 0.005-0.0172, range 0-0.0396)** | **0.0038 (IQR 0-0.0075, range 0-0.0201)** | **0.50 (0.23 to 0.69)** |
| **Heart rate features** | | | |
| Heart rate, mean (bpm) | 72.3 (IQR 67.7-78, range 64.2-85.8) | 76.7 (IQR 70.8-82.4, range 58.7-90.6) | -0.22 (-0.49 to 0.08) |
| Heart, sd (bpm) | 13.6 (IQR 12.6-15.5, range 9.6-18.6) | 12.9 (IQR 11.3-14.3, range 8-18) | 0.22 (-0.08 to 0.49) |
| Heart rate, minimum (bpm) | 53 (IQR 47-57.2, range 34-62) | 52.5 (IQR 50.4-59.5, range 44-71) | -0.23 (-0.50 to 0.07) |
| Heart rate, first quartile (bpm) | 62 (IQR 56.2-67.8, range 50-77) | 63.5 (IQR 59.9-71.9, range 51-84) | -0.21 (-0.48 to 0.10) |
| Heart rate, median (bpm) | 71 (IQR 66-77.5, range 59-83) | 73.5 (IQR 68-80.3, range 57-91) | -0.24 (-0.50 to 0.07) |
| Heart rate, third quartile (bpm) | 80 (IQR 76.4-86, range 70.5-92) | 85.5 (IQR 77-89.4, range 62.5-97.5) | -0.19 (-0.46 to 0.12) |
| Heart rate, maximum (bpm) | 127 (IQR 121.8-134, range 110-165) | 128.5 (IQR 120.9-137.9, range 99.5-148) | 0.01 (-0.29 to 0.31) |
| **Sleep features** | | | |
| Time asleep (min) | 382 (IQR 361-417.8, range 184-475.5) | 405 (IQR 375.8-424.2, range 292-495) | -0.15 (-0.43 to 0.16) |
| Time awake in bed (min) | 53.5 (IQR 46-60, range 7-80) | 58.5 (IQR 48.8-67.2, range 31-90) | -0.19 (-0.46 to 0.12) |
| Time total in bed (min) | 433.5 (IQR 406.2-491.5, range 198-539) | 468.5 (IQR 426-491.6, range 331-604) | -0.13 (-0.41 to 0.18) |
| Fitbit sleep efficiency (%) | 95 (IQR 93.8-96, range 51-98) | 94 (IQR 92-95, range 64-98) | 0.22 (-0.09 to 0.48) |
| Light sleep (min) | 236.5 (IQR 222.5-268.8, range 160-317) | 254 (IQR 236.2-288.4, range 177-331.5) | -0.24 (-0.50 to 0.06) |
| Deep sleep (min) | 68 (IQR 61.2-81.2, range 10.5-90) | 68.5 (IQR 61.5-78.5, range 31.5-98.5) | -0.03 (-0.33 to 0.27) |
| REM sleep (min) | 76 (IQR 65.8-90.5, range 42.5-119) | 77.5 (IQR 61.9-91, range 40.5-113) | 0.01 (-0.29 to 0.31) |

## Figure S1: Reliability overview: ICC vs mCV for all features and subtests


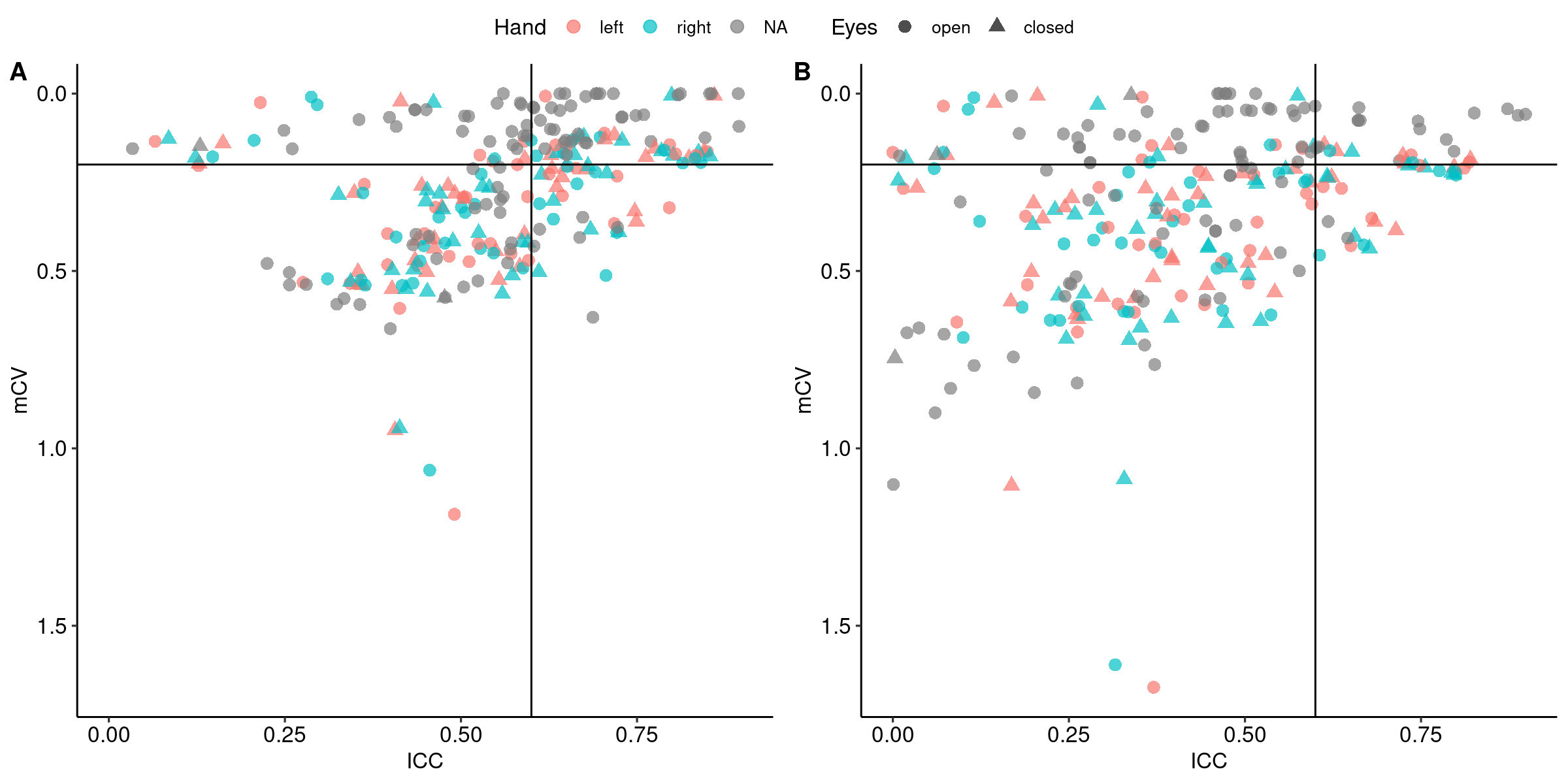


ICC: Intra Class Correlation (higher is better), mCV: median Coefficient of Variation (lower is better). (A) main analysis with exclusion of outliers, (B) sensitivity analysis with inclusion of outliers. Black lines represent the pre-defined cut-off values of ICC ≥ 0.6 or mCV < 0.2 resulting in 4 quadrants: the bottom left contains all features that did not meet our criteria, the bottom right contains features that met the ICC criterion only, the top left contains features that met the mCV criterion only, and the top right contains features that met both criteria. This figure shows all features and all subtests, resulting in a total of 256 points (compare with Table S4).

## Figure S2: Smartwatch median daily features in n=27 HV and n=28 PwMS


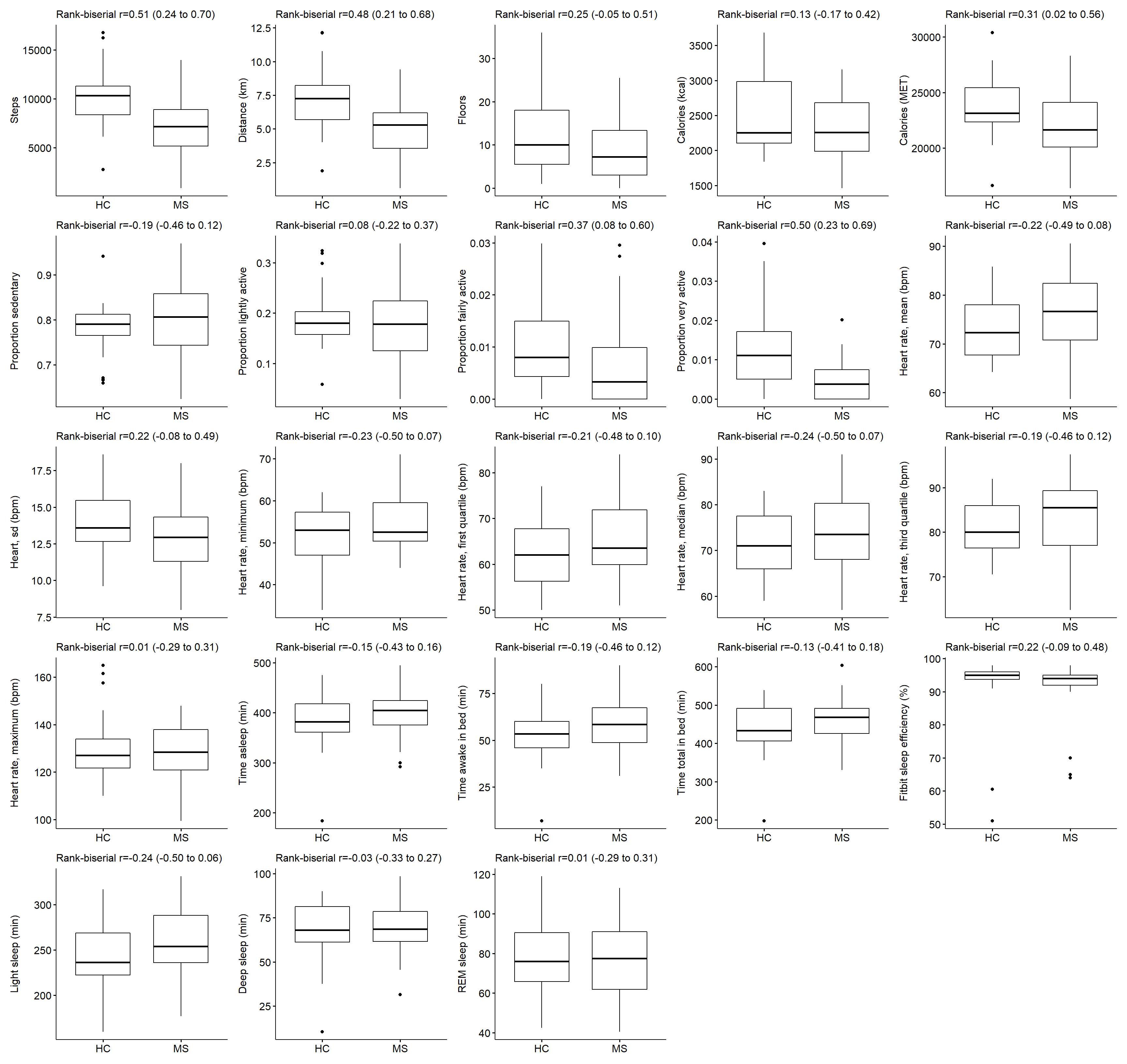


## Figure S3: Example screenshots from the dreaMS app version 1


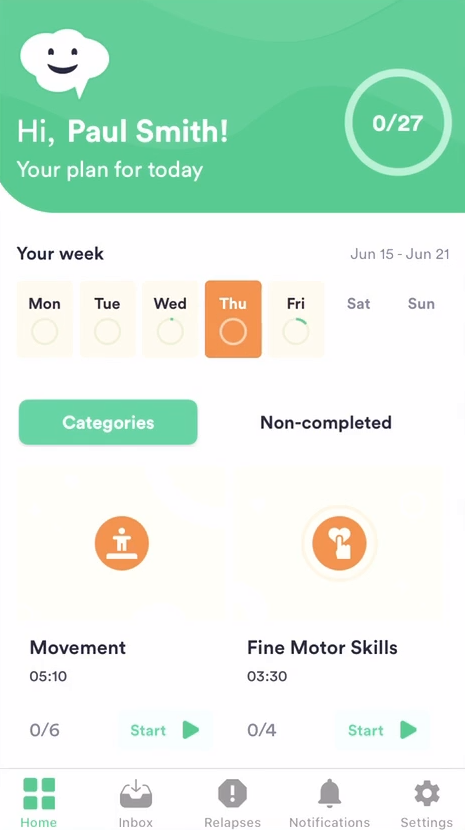

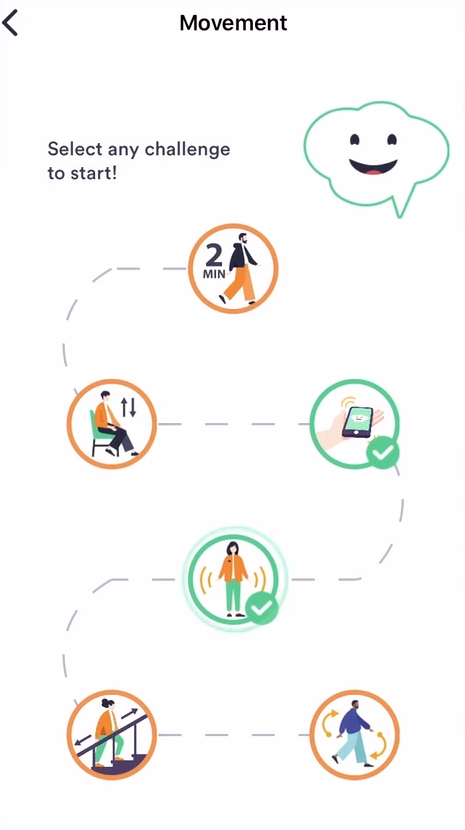

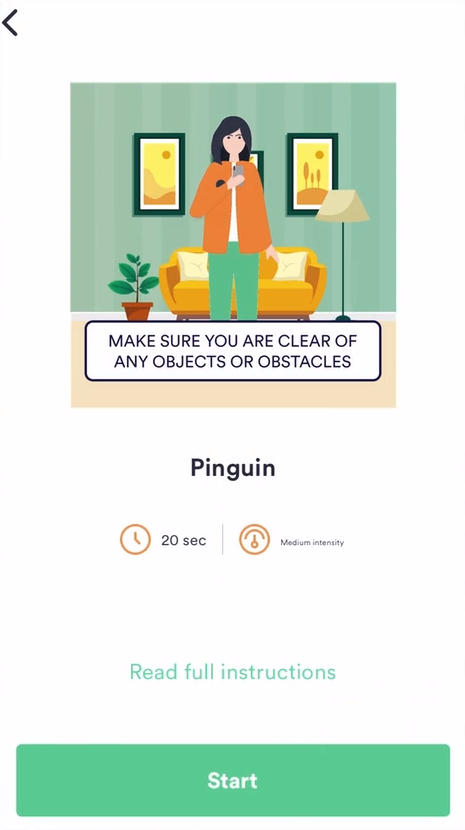

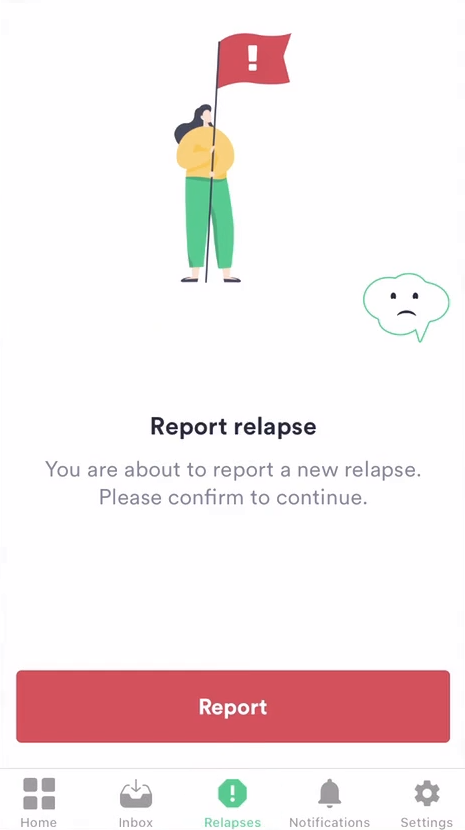

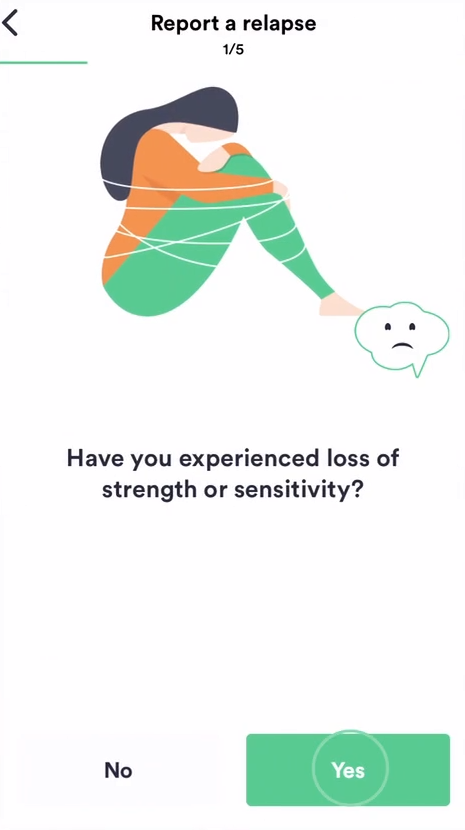

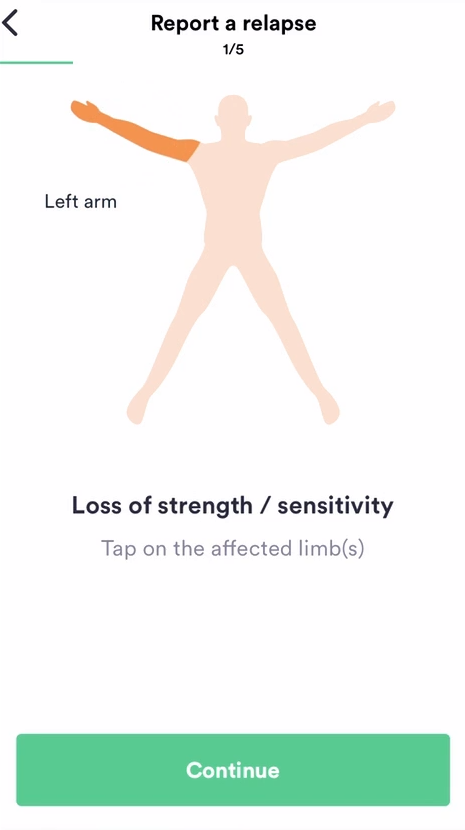

Supplement: Supplementary file 1 — Supplementary file1 (DOCX 1053 KB) [file 415_2022_11306_MOESM1_ESM.docx]
